# Cellular and Molecular Anatomy of the Human Neuromuscular Junction

**DOI:** 10.1016/j.celrep.2017.11.008

**Published:** 2017-11-28

**Authors:** Ross A. Jones, Carl Harrison, Samantha L. Eaton, Maica Llavero Hurtado, Laura C. Graham, Leena Alkhammash, Oladayo A. Oladiran, Andy Gale, Douglas J. Lamont, Hamish Simpson, Martin W. Simmen, Christian Soeller, Thomas M. Wishart, Thomas H. Gillingwater

**Affiliations:** 1 Edinburgh Medical School: Biomedical Sciences, University of Edinburgh, Edinburgh EH8 9AG, UK; 2 Euan MacDonald Centre for Motor Neurone Disease Research, University of Edinburgh, Edinburgh EH8 9AG, UK; 3 Physics and Astronomy, University of Exeter, Exeter EX4 4QL, UK; 4 Neurobiology, Roslin Institute, University of Edinburgh, Edinburgh EH25 9RG, UK; 5 Fingerprints Proteomics, University of Dundee, Dundee DD1 5EH, UK; 6 Department of Orthopaedic Surgery, University of Edinburgh, Edinburgh EH16 4SB, UK

**Keywords:** human, synapse, neuromuscular junction, aging, active zone, super-resolution imaging, proteomics, mouse, comparative anatomy, nervous system

## Abstract

The neuromuscular junction (NMJ) plays a fundamental role in transferring information from lower motor neuron to skeletal muscle to generate movement. It is also an experimentally accessible model synapse routinely studied in animal models to explore fundamental aspects of synaptic form and function. Here, we combined morphological techniques, super-resolution imaging, and proteomic profiling to reveal the detailed cellular and molecular architecture of the human NMJ. Human NMJs were significantly smaller, less complex, and more fragmented than mouse NMJs. In contrast to mice, human NMJs were also remarkably stable across the entire adult lifespan, showing no signs of age-related degeneration or remodeling. Super-resolution imaging and proteomic profiling revealed distinctive distribution of active zone proteins and differential expression of core synaptic proteins and molecular pathways at the human NMJ. Taken together, these findings reveal human-specific cellular and molecular features of the NMJ that distinguish them from comparable synapses in other mammalian species.

## Highlights

• Human neuromuscular junctions (NMJs) are morphologically distinct from rodent NMJs
• Human NMJs are remarkably stable across the adult lifespan
• Active zone proteins, including SNAP25, are differentially localized in human NMJs
• Significant divergence between the synaptic proteome of human and mouse NMJs

## Graphical Abstract

**Figure:**
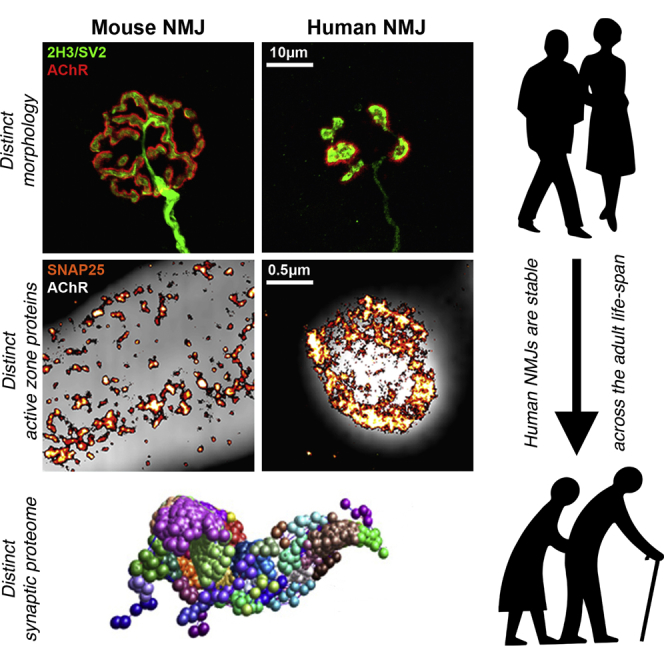

## Teaser

Jones et al. reveal fundamental differences between synapses in humans and lower mammals. They show that human neuromuscular junctions (NMJs) are smaller and more fragmented than comparable synapses from mice, with a distinct molecular composition. In contrast to mice, human NMJs were also remarkably stable across the entire adult lifespan.

## Introduction

Synapses play fundamental roles in the form and function of the nervous system both in health and during disease. Despite numerous important breakthroughs in our understanding of the cellular and molecular composition of synapses in animal models, both historic (Fatt and Katz, 1952, Couteaux and Pécot-Dechavassine, 1970, Nitkin et al., 1987) and recent (Oh et al., 2014, Wilhelm et al., 2014, Kasthuri et al., 2015), we know surprisingly little about the equivalent make up of synapses in humans. Current studies of synaptic connectivity at the cellular and molecular level have therefore relied heavily on “model” organisms, both vertebrate and invertebrate, working on the tacit assumption that the biological principles uncovered can ultimately be applied to humans.

The neuromuscular junction (NMJ) represents one major sub-class of synapse in the mammalian nervous system, critical for the transfer of information between the nervous system (lower motor neuron) and skeletal muscle. It also epitomizes a “model” synapse (Shi et al., 2012), both conveniently accessible within the peripheral nervous system and an early target in several neurodegenerative conditions, including amyotrophic lateral sclerosis and spinal muscular atrophy (Murray et al., 2010). Indeed, many of the fundamental principles governing synaptic form and function in the nervous system were discovered from early experiments examining NMJs in model organisms (Slater, 2015). More recently, the NMJ has been used to reveal core aspects of synaptic form and function *in vivo*, including the control of activity-dependent plasticity (Newman et al., 2017), as well as synaptic development and age-related decline (Liu et al., 2017).

Surprisingly, however, compared to extensive experimental data from animal models, there is currently a relative paucity of data concerning the cellular and molecular composition of the human NMJ. Ethical considerations and the logistics of obtaining biopsy material (in contrast to post-mortem sampling) from healthy individuals during life make it difficult to obtain tissue samples that are suitably well-preserved to facilitate high-resolution cellular and molecular analysis (Kay et al., 2013). Here, we report the development of a tissue harvesting and processing approach during surgical amputation that has allowed us to undertake a detailed cellular and molecular characterization of the healthy human NMJ across the adult lifespan.

## Results and Discussion

Tissue samples were obtained from surgical discard material from twenty patients undergoing lower limb amputation for a variety of clinical indications (for full patient details, see Experimental Procedures; summarized in Table S2), including complications of peripheral vascular disease (PVD) and non-PVD-related cases (e.g., for chronic pain following previous orthopedic surgery or chronic osteomyelitis refractory to antibiotic treatment). Importantly, samples were obtained from non-pathological—otherwise healthy—regions of limb (e.g., close to the site of amputation) where the tissue needs to be free from any disease or pathology in order for sufficient post-operative tissue healing to occur. NMJs from four anatomically distinct muscles were harvested: *extensor digitorum longus* (EDL), *soleus* (S), *peroneus longus* (PL), and *peroneus brevis* (PB). For comparison, the same muscles were dissected from a single litter of young adult CD1 mice. NMJs were immunohistochemically labeled, imaged, and analyzed using a standardized platform: “NMJ-morph” (Jones et al., 2016). For each NMJ, 21 individual synaptic variables were measured. Baseline data were obtained for 2,860 human NMJs across seven decades of life (from the ages of 34–92 years).

### Cellular Architecture of the Human NMJ

**Figure 1:**
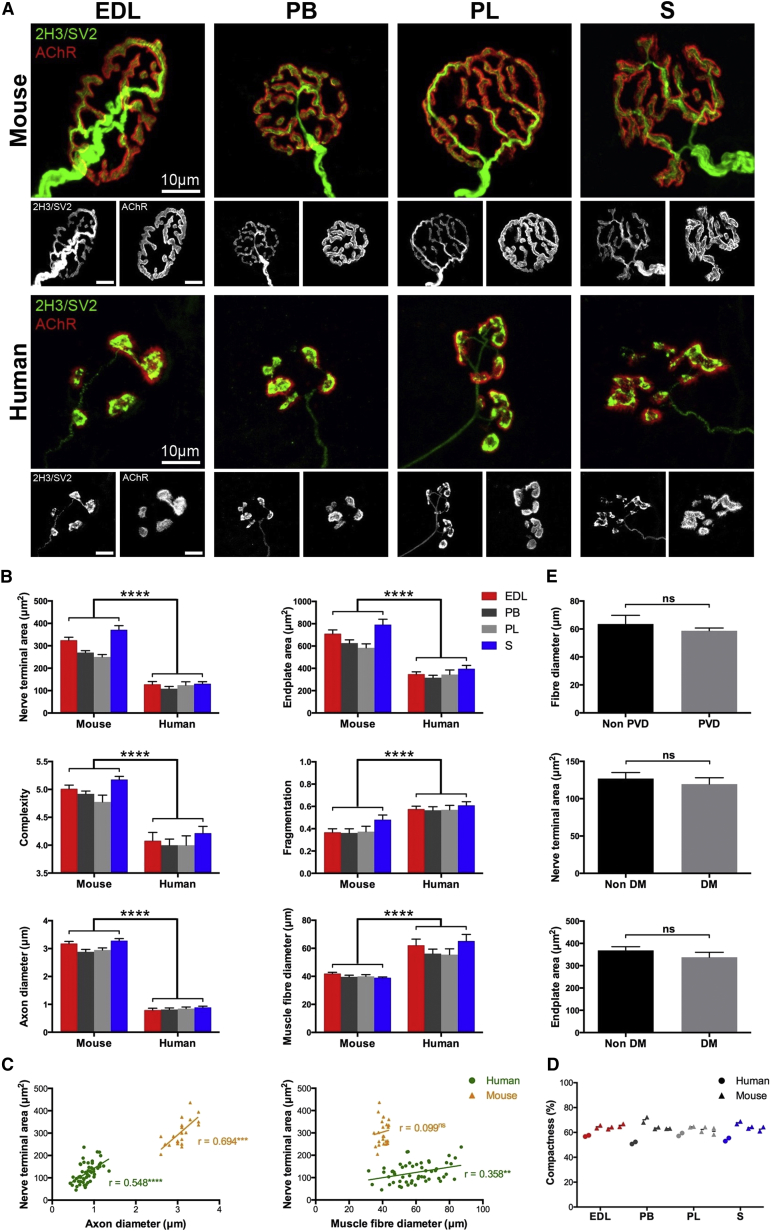
Unique Morphology of the Human NMJ (A) Representative confocal micrographs from 4 lower limb muscles. Human NMJs are smaller, with a thinner axon, less complex nerve terminals and a distinctive “nummular” endplate. Scale bar, 10 μm. (B) Bar charts demonstrating significant species-specific differences across a range of pre- and post-synaptic variables. Each bar represents the mean (±SEM) of >600 human NMJs (and 240 mouse NMJs). Unpaired t test and Mann-Whitney test. (C) Scatterplots showing correlation between nerve terminal area and axon/muscle fiber diameter. Each data point is an individual muscle (mean of 40 NMJs) (72 human and 24 mouse muscles). NMJ morphology was more closely correlated with structural features of the pre-synaptic cell (motor axon) in both species. Pearson correlation. (D) Left/right muscles pairs from the same human case were compared with 3 mouse controls. Each data point (in the pair) is an individual muscle (left or right); the intervening line is the mean of the 2 sides. Laterality did not influence NMJ morphology in either species. (E). Effect of co-morbidities on human NMJ morphology. No significant morphological differences could be attributed to either diabetes mellitus (DM) or peripheral vascular disease (PVD). Unpaired t tests. ^∗∗∗∗^p < 0.0001, ^∗∗∗^p < 0.001, ^∗∗^p < 0.01, ^∗^p < 0.05.

Initial qualitative observations revealed striking morphological differences between human and mouse NMJs (Figure 1). Human NMJs were universally smaller than their mouse counterparts, with much thinner pre-terminal axons, more rudimentary nerve terminals, and distinctive “nummular” (formed of coin-shaped patches) endplates (Figure 1). These observations were confirmed quantitatively (Table S1). Axon diameter and average area of AChR clusters showed the greatest differences between species (3.69-fold^∗∗∗∗^ and 3.33-fold^∗∗∗∗^, respectively; higher in mice), with over half of the morphological variables studied (12 of 21) showing a fold difference of at least 150% between humans and mice. The degree of overlap between pre- and post-synaptic components (the proportion of AChR labeling at the motor endplate that is directly covered by overlying motor nerve terminal) was relatively similar between the species (50% in humans, 64% in mice), in agreement with previous electron microscopy (EM) studies (Slater et al., 1992). None of the human NMJs analyzed lacked pre-synaptic boutons overlying AChR clusters (Figure 1); the only difference was the degree to which AChRs were dispersed beyond the limits of the nerve terminals. Thus, each and every individual AChR island at the motor endplate was innervated by a corresponding motor nerve terminal bouton. Importantly, no significant morphological differences at the human NMJ could be attributed to patient co-morbidities (diabetes mellitus, vascular disease) (Figure 1; also see Experimental Procedures), and the side of the body examined did not influence morphology (Figure 1), consistent with our previous findings in mice (Jones et al., 2016).

Although human NMJs were routinely only half the size of their mouse counterparts, with axons only a third of the caliber, they were found to innervate muscle fibers up to twice the diameter of those in mice (Figure 1). We therefore assessed the relationship between NMJ morphology and pre- and post-synaptic cells by correlating each morphological variable with motor axon diameter and muscle fiber diameter respectively. In both species, NMJ morphology correlated more strongly with motor axon diameter (Jones et al., 2016) (Figure 1; Table S1). Correlation coefficients (r) were higher in relation to axon diameter for the majority of NMJ variables in both species (10 of the 18), with only a minority (6 in humans, 1 in mice) being more closely correlated with the muscle fiber, suggesting that the morphological properties of the motor neuron (as determined by measuring axon diameter) exerted a stronger influence on synaptic morphology in humans than morphological properties of the skeletal muscle fiber.

To confirm that the differences in NMJ morphology we observed between humans and mice were a consistent observation between humans and other mammals, we also compared our human and mouse data with NMJs from adult rats (Figure S1). Here, NMJ morphology was virtually indistinguishable between mice and rats across all muscles examined, with both species being clearly distinctive from humans. In addition, this comparison allowed us to establish whether the relatively small size of human NMJs is simply a consequence of increased body size. As an adult rat is considerably larger than a comparable mouse (∼10-fold increase in body weight), if NMJ size was inversely correlated with body size, we would have expected rat NMJs to be notably smaller than mouse NMJs (e.g., an intermediate NMJ size, somewhere between mice and humans). As NMJs from mice and rats were morphologically indistinguishable from each other, we conclude that the smaller size of human NMJs cannot simply be a consequence of increased body size and mass (Figure S1).

Taken together, these findings reveal that human NMJs have a unique morphology, being significantly smaller and more fragmented than comparable synapses from widely used animal models (mice and rats). This challenges the simplistic assumption that structures in the human nervous system are inevitably larger and more complex than those in lower mammals.

### The Human NMJ across the Lifespan

One area of research that is currently receiving significant interest concerns an apparent age-related decline in synaptic stability at the NMJ, manifesting as degenerative changes affecting both the pre-synaptic motor nerve terminal and the post-synaptic motor endplate (Gonzalez-Freire et al., 2014, Liu et al., 2017). Although findings from animal models (Anis and Robbins, 1987, Balice-Gordon and Lichtman, 1990, Valdez et al., 2010, Willadt et al., 2016) suggest that NMJs are inherently unstable with age, it is unclear whether a similar phenomenon occurs across the longer human lifespan. We were able to address this important question as our human tissue samples incorporated patients from the fourth to the tenth decades of life, with the individual ages of patients distributed approximately evenly across the age range (Table S2).

**Figure 2:**
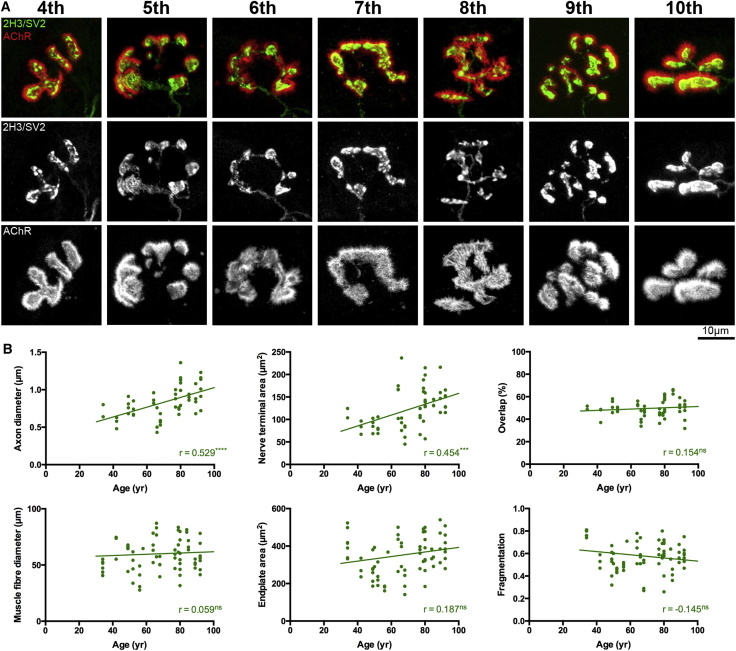
The Human NMJ Is Stable across the Lifespan (A) Representative confocal micrographs of human NMJs from the 4th to the 10th decades of life (all from *peroneus longus* muscle). Despite the heterogeneity of individual NMJs, the overall appearance is conserved across the 70+ year age range. Scale bar, 10 μm. (B) Scatterplots showing correlations between age and a range of individual pre- and post-synaptic NMJ variables. Data are pooled across the 4 muscle groups (72 individual muscles). Each data point is an individual muscle (mean of 40 NMJs). Although 2 of the pre-synaptic variables shown correlated with age (a modest increase in the size of the pre-synaptic axon and motor nerve terminal), overall synaptic morphology remained remarkably stable. Pearson and Spearman correlation. ^∗∗∗∗^p < 0.0001, ^∗∗∗^p < 0.001.

Qualitative analyses of NMJs suggested conservation of synaptic structure across the entire lifespan in humans (Figure 2). The only change observed with increasing age was a modest increase in axon diameter (r = 0.529^∗∗∗∗^) (Figure 2). Notably, there was no significant change in muscle fiber diameter or endplate area with age. Nor were there any age-associated changes in the degree of overlap between pre- and post-synaptic components of the NMJ, or any evidence for age-associated fragmentation of the NMJ (Figure 2). Furthermore, the modest degree of pre-synaptic remodeling that occurred with age was de-coupled from equivalent changes in the muscle fiber, further supporting our finding that synaptic morphology is more closely correlated with the pre-synaptic neuron. Thus, the human NMJ remains remarkably stable across the adult lifespan, devoid of any of the age-related degeneration and/or remodeling changes that have been reported in other mammalian species occurring over a much shorter time scale (Valdez et al., 2010, Willadt et al., 2016).

Interestingly, these findings partially contradict an earlier study suggesting that the human NMJ undergoes changes over the adult lifespan (Oda, 1984). These differences are most likely explained by methodological disparities between the studies and a smaller sample size in the Oda (1984) study. For example, Oda used relatively low-resolution techniques (e.g., silver and cholinesterase staining) to study 12 autopsy samples obtained 6 hr after death. Our own preliminary experiments confirmed that we needed to harvest tissue quickly (e.g., within minutes) from freshly biopsied material (not post-mortem) in order to obtain accurate morphological measurements. Moreover, our findings are in agreement with another smaller study of human muscle samples where endplate size was found to remain stable with age (Wokke et al., 1990).

### Super-Resolution (Direct Stochastic Optical Reconstruction Microscopy) Imaging of Active Zone Proteins at the Mouse and Human NMJ

Given the structural differences we observed between human and mouse NMJs, we next wanted to establish whether human NMJs were also distinct from the molecular perspective. In our initial morphological experiments, labeling of the synaptic vesicle protein SV2 appeared to be qualitatively different between human and mouse nerve terminals. Synaptic boutons in mice were characterized by relative homogeneity of labeling, whereas motor nerve terminals at human NMJs contained distinctive “hotspots” of fluorescence (particularly clear examples can be seen in the 2H3/SV2 greyscale panels for the 4^th^, 7^th^, and 9^th^ decade images shown in Figure 2). Because the functional architecture of active zones varies considerably between species (Ackermann et al., 2015), we hypothesized that the heterogeneous labeling of SV2 in humans reflected differential spatial arrangement of active zone proteins/material at human synapses.

We assessed active zone protein distribution in human NMJs by performing direct stochastic optical reconstruction microscopy (dSTORM) super-resolution imaging (Huang et al., 2010) of one key active zone protein (SNAP25). SNAP25 is a component of the SNARE complex, a series of structural proteins responsible for the fusion of synaptic vesicles with the plasma membrane during exocytosis (Han et al., 2017), that is also known to be localized to active zones *in vivo* (Wilhelm et al., 2014). dSTORM imaging at the mouse NMJ revealed a remarkably similar punctate distribution of SNAP25 to that previously reported from studies of other active zone proteins in a variety of species using a range of different techniques (Fukunaga et al., 1983, Meinertzhagen et al., 1998, Nagwaney et al., 2009). For example, quantitative comparisons between our mouse SNAP25 data and recent data from STED microscopy experiments studying the active zone proteins Bassoon and Piccolo in mice (Nishimune et al., 2016) revealed remarkably similar subcellular distribution: the average density of Bassoon and Piccolo “puncta” in the Nishimune et al. (2016) study was ∼10 per μm^2^, with similar analyses of SNAP25 in our study revealing a density of ∼15 per μm^2^.

**Figure 3:**
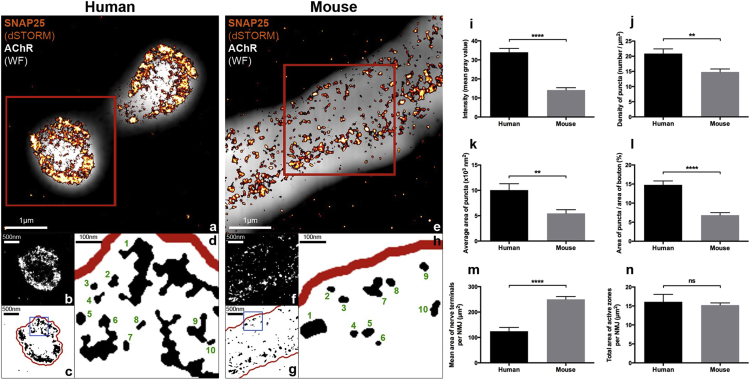
Comparative Super-Resolution (dSTORM) Imaging of the Active Zone Protein SNAP25 at Human and Mouse NMJs (A and E) Composite images of SNAP25-labeled nerve terminals (dSTORM, orange/red) overlaid on BTX-labeled AChRs of the motor endplate (wide-field, gray). Note that (A) only shows two single synaptic boutons from the human NMJ (not the whole NMJ), and (B) only shows a sub-region of one single synaptic bouton from the mouse NMJ (again not the whole NMJ). Scale bars, 1 μm. (B and F) 8-bit greyscale images of single boutons (representing the areas contained within the red boxes in A and E) used to quantify intensity of labeling (I). Note the increased intensity and density of SNAP25 in the human NMJ. Scale bars, 500 nm. (C and G) Despeckled, binary versions of (B) and (F) used to quantify the remaining variables (J–L). The boxed areas have been enlarged (D and H) to depict individual SNAP25 puncta; 10 discrete puncta have been labeled in each image. Scale bars, 100 nm. (I–L) Bar charts showing dSTORM image quantification of SNAP25 intensity (I), density of SNAP25 puncta (J), average area of SNAP25 puncta (K), and the area of SNAP25 puncta as a percentage of total bouton area (L), at human and mouse NMJs. All 4 measures of SNAP25 labeling were significantly greater in the human NMJs. For both human and mouse datasets, n = 50 boutons; bar charts depict mean (±SEM). Unpaired t test and Mann-Whitney test. (M and N) Bar charts comparing the average size of human and mouse NMJs (nerve terminal area, M) with the total size of their active zone material (SNAP25 area per NMJ, N). Although the human NMJ is significantly smaller than the mouse NMJ (M), the total amount of SNAP25 labeling at the NMJ is the same when adjusted to reflect the total overall size of the synapse (N). Unpaired t test. ^∗∗∗∗^p < 0.0001, ^∗∗^p < 0.01.

The finding that dSTORM imaging could be used to reliably label and quantify SNAP25 distribution at the mouse NMJ prompted us to apply dSTORM imaging to compare SNAP25 localization between mouse and human NMJs. Parallel dSTORM imaging of human and mouse NMJs revealed clear differences in the distribution and intensity of SNAP25 between the two species (Figure 3). We quantified a total of 2,945 (human) and 10,666 (mouse) individual SNAP25 puncta, from 50 boutons (10 NMJs) of 3 individual patients/mice. All four core variables measured were found to be significantly greater in humans than in mice: the average area of individual SNAP25 puncta and their density within each bouton (both ^∗∗^p < 0.01), the total area of all puncta relative to that of each bouton (^∗∗∗∗^p < 0.0001), and the intensity of SNAP25 labeling (^∗∗∗∗^p < 0.0001).

Given that motor nerve terminals (and hence the “area of synaptic contact”) at the human NMJ are significantly smaller than those in mice (Figure 1; Table S1), we calculated the total area of SNAP25 labeling per NMJ. This analysis revealed the total area to be identical in both humans and mice: approximately 15 μm^2^ of SNAP25 per NMJ (Figure 3). Therefore, although the human NMJ is significantly smaller than the mouse NMJ, the total amount of SNAP25 protein (and, by extension, the amount of molecular active zone machinery) is similar between human and mouse NMJs, albeit packaged into significantly smaller synaptic boutons in humans. This observation suggests the possible presence of a homeostatic mechanism that preserves the functional architecture of the synapse in the face of significant morphological heterogeneity. In conjunction with the extensive post-synaptic junctional folding demonstrated previously (Slater et al., 1992), this pre-synaptic specialization could play a role in effectively maintaining neurotransmission at the smaller human NMJ. The application of super-resolution imaging to visualize active zone proteins at the human NMJ reported here paves the way for future studies that will be able to develop a more refined and detailed subcellular “map” of synaptic protein distribution and localization across different species. Such studies may also assist in addressing the apparent discrepancies that exist between the species-specific differences we report with respect to the molecular composition of active zones and previous freeze-fracture studies that suggested more consistent conservation of gross active zone structure between human and rodent synapses (for review see Rogers and Nishimune, 2017). Of note, we were unable to reliably label human NMJs with antibodies against Bassoon or Piccolo, which may point to species-specific differences in these antigens.

### Molecular Profiling of the Human NMJ

**Figure 4:**
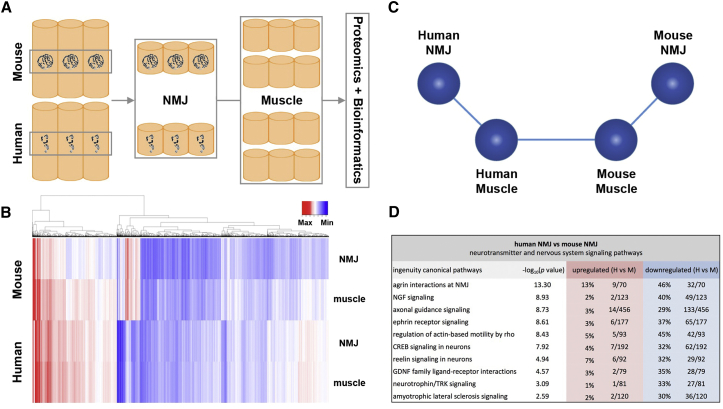
Unique Molecular Profile of the Human NMJ (A) Schematic representation of sample micro-dissection used to produce NMJ-enriched and NMJ-devoid (muscle) samples for proteomics and subsequent bioinformatics analysis. NMJ presence/absence was confirmed by α-bungarotoxin labeling (Experimental Procedures). (B) Heatmap representation of proteomic data. Abundance is indicated by color intensity, from lowest (darkest blue) to highest (darkest red). Clear differences exist between the 4 samples, particularly when comparing human and mouse NMJs. (C) Schematic representation of proteomic data based on Biolayout output (Experimental Procedures). Each sphere represents the entire expression data for an individual tissue sample, and the proximity and orientation relative to one another indicates the similarity of the datasets; human and mouse muscle samples are more similar than human and mouse NMJ samples. (D) *In silico* analysis identifies canonical cascades relating to neurotransmitter function and nervous system signaling. Ten example pathways are listed. Values are the percentage (and number) of proteins in each cascade that are upregulated (red) or downregulated (blue) in human cf. mouse. Significance levels are listed as –log_10_(p value), i.e., –log_10_(p < 0.05) ≈1.3, –log_10_(p < 0.0001) = 4, etc. Of the 36 pathways identified, only 1 showed no significant species difference (GABA receptor signaling cascade; not shown).

To investigate potential differences in the broader species-specific molecular composition of the human NMJ, we next utilized state-of-the-art proteomic techniques (tandem mass tagging) to undertake comprehensive proteome-wide profiling of human and mouse NMJs. By establishing the proteomic profile of micro-dissected NMJ-enriched and NMJ-devoid skeletal muscle samples (Figures 4 and S2), we were able to establish and compare the protein-level composition of NMJs and muscle fibers in human and mouse samples. Through a combined human/mouse database search we confirmed the identification of peptides associated with 6,737 proteins. Application of stringent filtering parameters in order to ensure reliability of protein identification subsequently yielded 5,026 proteins for bioinformatics analyses. This represents a high level of coverage from a single proteomic analysis and compares favorably with a recent database of human muscle proteins identified by mass spectrometry that lists 5,431 muscle proteins across 38 peer reviewed scientific publications from 2002–2017 (Gonzalez-Freire et al., 2017). Moreover, we were able to demonstrate the identification of synaptic and neuronal proteins within our NMJ-enriched dissections (Figures S3 and S4).

Comparative bioinformatics analysis of proteomic profiles from human and mouse skeletal muscle samples (devoid of NMJs) revealed clear molecular overlap (Figure 4; Table S3), with the majority (66%) of the 200 known metabolic cascades identified showing no significant species-dependent differences. In contrast, the expression profiles for the NMJ-enriched samples indicated a clear molecular variation between humans and mice (Figure 4). For example, *in silico* analysis identified 36 distinct nervous system-related molecular pathways known to impact on NMJ form and function (including a range of core synaptic signaling pathways, such as “integrin signaling,” “axonal guidance signaling,” and “CREB signaling in neurons”) where proteins were present in both human and mouse datasets. Surprisingly, 97% of these pathways showed statistically significant differences in protein expression levels between the two species (Figures 4 and S4). For example, major differences were observed in the levels of 24 individual proteins contributing to agrin signaling pathways at the NMJ (Figure S4). Importantly, each affected pathway included some proteins that were more abundant in human samples as well as other proteins that were more abundant in mouse samples, confirming that the differences observed were not simply an artifact of relative enrichment during tissue processing. Thus, these proteomics datasets provide evidence to suggest that human NMJs have a significantly modified molecular composition compared to analogous NMJs in mice. This finding is consistent with recent observations of species-specific gene expression in developing neurons from humans and mice (Qiu et al., 2016).

Taken together, our findings reveal human-specific features of the NMJ that distinguish them from comparable synapses in other mammalian species. These fundamental differences between synapses in humans and lower mammals must be taken into careful consideration when interpreting animal-based studies with respect to their applicability to humans.

## Experimental Procedures

Further details and an outline of resources used in this work can be found in Supplemental Experimental Procedures.

### Ethics

Use of anonymous human tissue was granted by the Lothian NRS BioResource (SR719, 15/ES/0094); prospective tissue collection was approved by the Lothian Ethics Committee (REC 2002/1/22, 2002/R/OST/02) following internal (University of Edinburgh) and independent/external ethical review.

### Human Case Series

Human muscle samples were obtained from patients following lower limb amputation surgery (see above for ethical/institutional approvals). In total, 21 sets of muscle samples were obtained from 20 patients (15 male, 5 female)—1 patient required a bilateral procedure. The clinical details for each patient are summarized in Table S2. The majority of patients (16 out of 20) underwent amputation for complications of peripheral vascular disease (PVD), typically either critical ischemia in a non-salvageable limb, or failure of previous vascular reconstruction. Most of these cases (12 out of 16) were below-knee amputations (BKA); only four patients required above-knee amputation (AKA). Of the four non-PVD-related cases, BKA was performed in two patients for chronic pain following previous orthopedic surgery (49F, 50M), in one patient for chronic osteomyelitis refractory to antibiotic treatment (42F) and in a final patient (34M) who required bilateral amputation for acute ischemia (secondary to thromboembolism from infective endocarditis). Mean age at surgery was 67 years (range 34–92).

The choice of muscles was primarily dictated by the logistics and reproducibility of sampling, given the variation in level and quantity of discard material for each amputation, but with a view to including a range of muscle types (fast, slow, mixed). The original technique of motor point biopsy (for the peroneal muscles) as described by Coërs and Woolf (1959) was used as a guide. For most of the case series, we were able to obtain a complete set of samples from the four muscles chosen. In total, 72 individual muscles were analyzed (EDL = 19, S = 18, PB = 18, PL = 17).

### Mice and Rats

To allow direct comparison with human NMJs, equivalent muscles were dissected from both sides of three CD1 littermate mice (adult, ∼12 weeks old) and wild-type rats (adult, ∼16 weeks old). Animals were euthanized with isoflurane and the muscles dissected out within 30 min post-mortem and fixed in 4% PFA for 1 hr. All animal experiments were performed under the appropriate project and personal licenses granted by the UK Home Office.

### Statistical Analysis

All statistical analyses were performed in GraphPad Prism. Individual statistical tests and significance levels are referred to in the relevant text sections and corresponding figure legends.

## Author Contributions

R.A.J. and T.H.G. conceived and designed the study. R.A.J., C.H., C.S., S.L.E., T.M.W., and T.H.G. planned experiments. R.A.J., C.H., S.L.E., M.L.H., L.C.G., L.A., O.A.O., A.G., D.J.L., M.W.S., C.S., T.M.W., and T.H.G. performed experiments and data analysis. All authors drafted and approved the manuscript for submission.

## Data and Software Availability

The full raw proteomics data files from this study are freely available for download from: https://datashare.is.ed.ac.uk/handle/10283/2937.

## Supplemental Information

Document S1. Supplemental Experimental Procedures, Figures S1–S4, and Tables S1–S3

Document S2. Article plus Supplemental Information
