# Supplementary material for: Cellular and Molecular Anatomy of the Human Neuromuscular Junction
Source: Cell Rep. 2017 Nov 28;21(9):2348–56. doi: 10.1016/j.celrep.2017.11.008 (PMC5723673; doi:10.1016/j.celrep.2017.11.008)
Supplement: Document S1. Supplemental Experimental Procedures, Figures S1–S4, and Tables S1–S3 [file mmc1.pdf]

Cell Reports, Volume *21*

Supplemental Information

Cellular and Molecular Anatomy of the Human Neuromuscular Junction

Ross A. Jones, Carl Harrison, Samantha L. Eaton, Maica Llavero Hurtado, Laura C. Graham, Leena Alkhammash, Oladayo A. Oladiran, Andy Gale, Douglas J. Lamont, Hamish Simpson, Martin W. Simmen, Christian Soeller, Thomas M. Wishart, and Thomas H. Gillingwater

Figure S1

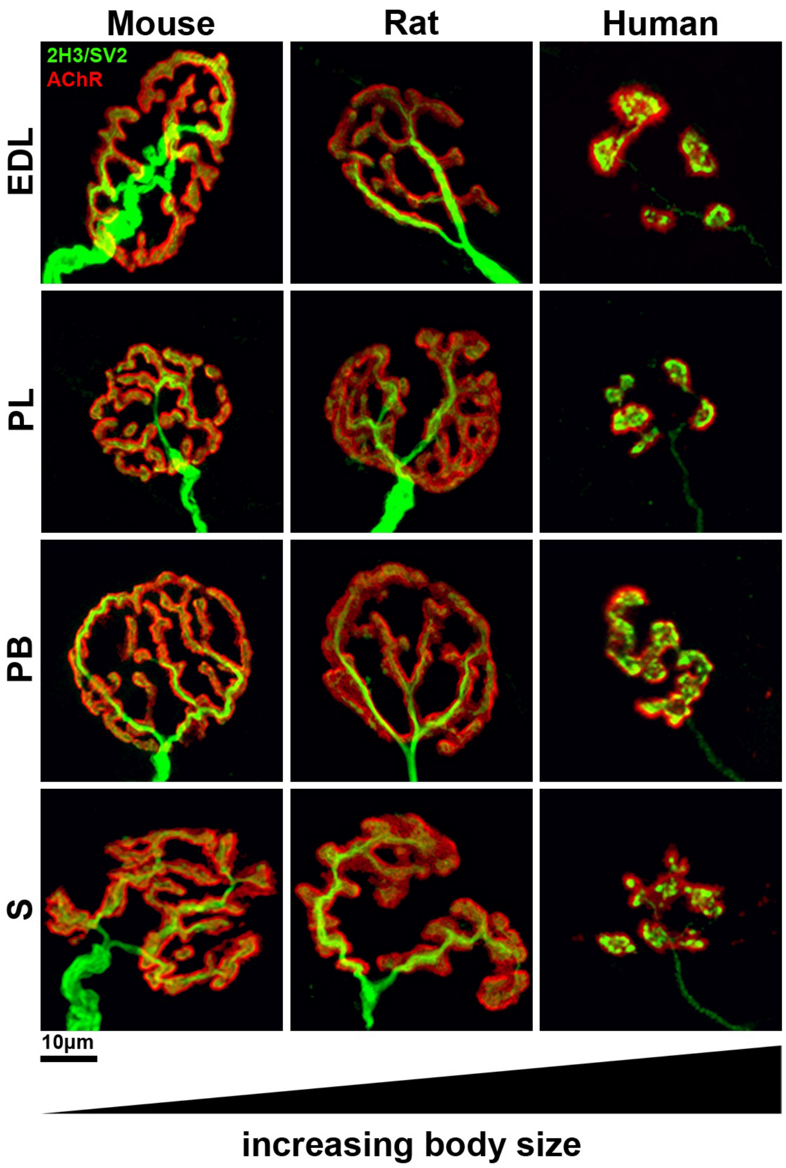

Figure S1 | Comparative anatomy of mouse, rat and human NMJs, Related to Figure 1.

Representative confocal micrographs of NMJs from equivalent muscles in the 3 different species, arranged from left to right in order of ascending body weight and size. There were no discernable differences between mouse and rat NMJs. Scale bar = 10 μm.

Figure S2

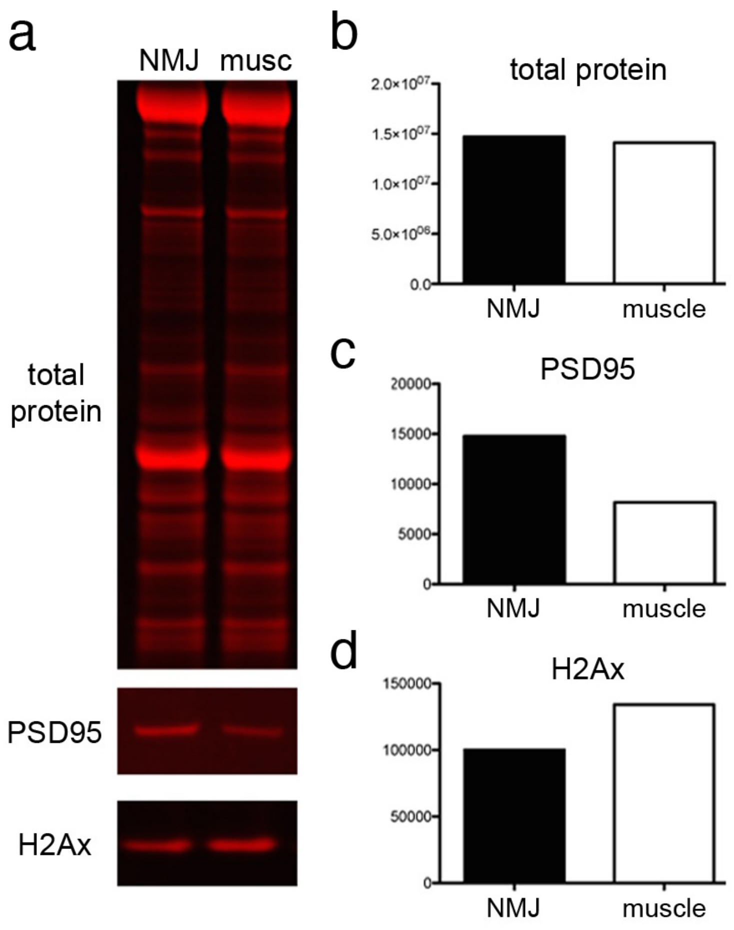

Figure S2 | Validation of NMJ-enrichment in human and mouse tissue, Related to Figure 4.

a. Samples enriched for NMJ content (NMJ) and devoid of NMJ content (muscle) were probed to confirm the efficacy of the isolation procedure used (see Methods and Figure 4). Representative LICOR captured images of gels stained for total protein load, and membranes probed for a post-synaptic marker (PSD95) and a nuclear marker (H2Ax) confirming **b.** equivalent protein extraction, **c.** enrichment for post-synaptic protein and **d.** de-enrichment for nuclei.

Figure S3

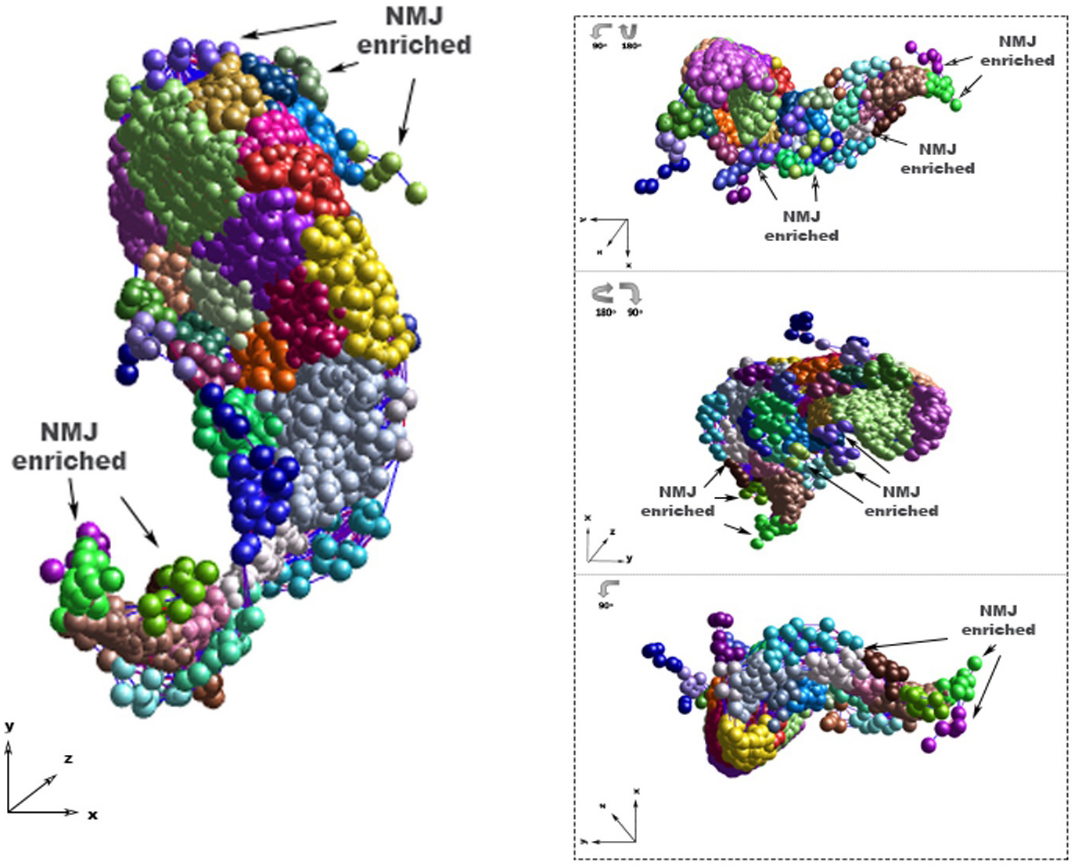

Figure S3 | Biolayout expression profile clustering identifies upregulated synaptic proteins in NMJ-enriched tissue samples, Related to Figure 4.

Nodes (spheres) represent individual proteins and edges (lines) reflect the strength of correlation (of expression) between proteins. Node colours represent distinct protein clusters based on their expression profile. The 3D schematic (shown in 4 different orientations) displays all proteins from both NMJ-enriched and NMJ-devoid human samples; the 5 distinct synaptic protein clusters from the NMJ-enriched samples are indicated with arrows.

Figure S4

Agrin interactions at the NMJ
(human vs mouse)

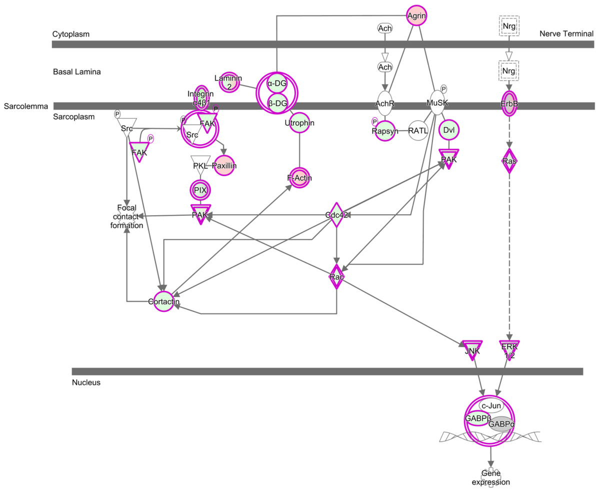

Axonal guidance signaling
(human vs mouse)

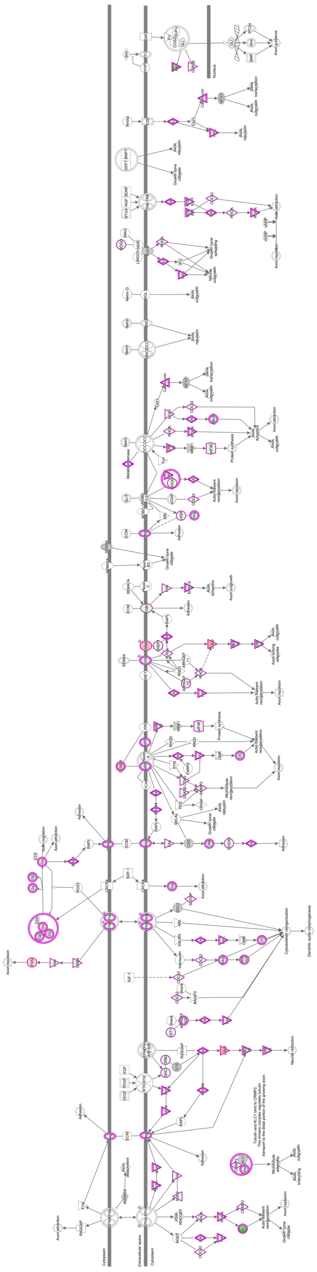

CREB signaling in neurons
(human vs mouse)

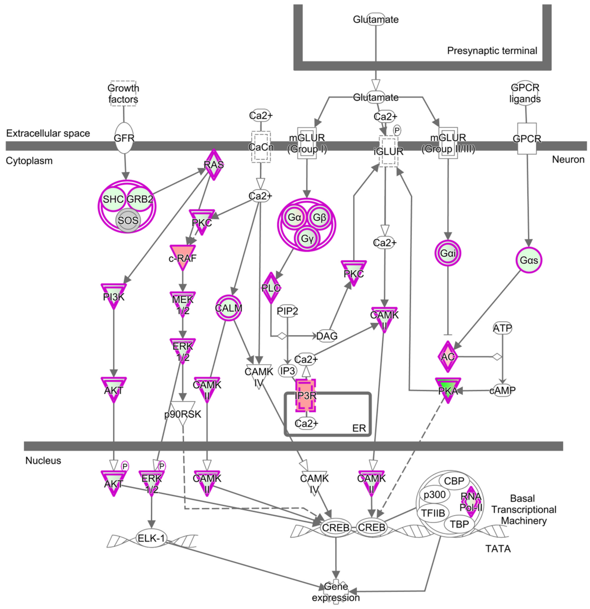

Figure S4 | Differences in protein composition between human and mouse NMJs, Related to Figure 4.

IPA canonical pathway analysis of proteomics data identified significant alterations in the molecular composition of NMJ-enriched samples from humans compared with mice. Several changes in core synaptic pathways such as agrin interactions at the NMJ (**a**), CREB signaling in neurons (**b**) and axonal guidance signaling (**c**) were identified. Nodes in red represent proteins more abundant in human samples relative to mouse (>20% increased in human); nodes in green represent proteins more abundant in mouse samples relative to human (>20% decreased in human); the darker the colour of the node, the greater the difference in protein expression. Grey nodes represent proteins that differ by <20% between human and mouse. Note that each pathway includes proteins that are both *more and less* abundant in human cf. mouse samples, indicating that this is not simply an artefact of relative enrichment during tissue processing.

Table S1

Table S1 Baseline morphological data and influence of pre- and post-synaptic cells

	average human NMJ	average mouse NMJ	fold difference	human NMJ: correlation with axon diameter	human NMJ: correlation with muscle fibre diameter	mouse NMJ: correlation with axon diameter	mouse NMJ: correlation with muscle fibre diameter
core varaibles							
pre-synaptic							
**1) nerve terminal area (µm^2)**	122.7	304.0	2.48 ****	0.548 ****	0.358 **	0.694 ***	0.099
**2) nerve terminal perimeter (µm)**	151.1	327.4	2.17 ****	0.435 **	0.408 **	0.429 *	0.273
**3) number of terminal branches**	28.0	30.0	1.07	0.248	0.355 **	0.370	0.016
**4) number of branch points**	14.0	26.0	1.86 ****	0.553 ****	0.250	0.609 **	-0.025
**5) total length of branches (µm)**	69.3	166.7	2.41 ****	0.533 ****	0.320 *	0.577 **	0.193
post-synaptic							
**6) AChR area (µm^2)**	206.7	424.2	2.05 ****	0.329 *	0.385 ***	0.572 **	0.076
**7) AChR perimeter (µm)**	152.2	275.8	1.81 ****	0.179	0.297 *	0.279	0.378
**8) endplate area (µm^2)**	351.5	678.2	1.93 ****	0.302 *	0.373 **	0.510 *	0.158
**9) endplate perimeter (µm)**	98.7	118.8	1.20 ****	0.363 **	0.373 **	0.521 **	0.060
**10) endplate diameter (µm)**	36.0	42.2	1.17 ***	0.350 *	0.344 **	0.421 *	0.069
**11) number of AChR clusters**	3.9	2.6	1.50 ****	0.225	0.151	0.306	0.181
derived variables							
pre-synaptic							
**12) average length of branches (µm)**	3.0	6.7	2.23 ****	0.522 ****	-0.146	-0.397	0.114
**13) complexity**	4.07	4.97	1.22 ****	0.424 **	0.275 *	0.582 **	0.064
post-synaptic							
**14) average area of AChR clusters (µm^2)**	71.7	238.5	3.33 ****	0.202	0.305 **	0.117	-0.085
**15) fragmentation**	0.58	0.40	1.45 ****	0.304 *	0.089	0.353	0.175
**16) compactness (%)**	61.6	64.4	1.05 **	0.000	-0.025	0.097	-0.357
**17) overlap (%)**	49.6	64.2	1.29 ****	0.382 **	-0.129	0.359	0.454 *
**18) area of synaptic contact (µm^2)**	105.2	267.9	2.55 ****	0.461 ***	0.370 **	0.660 ***	0.147
associated nerve & muscle variables							
**19) axon diameter (µm)**	0.84	3.10	3.69 ****				
**20) muscle fibre diameter (µm)**	59.9	40.2	1.49 ****				
**21) number of axonal inputs**	1	1	1.00				

Table S1 | Baseline morphological data and influence of pre- and post-synaptic cells, Related to Figure 1.

Core variables (1-11) are listed in red, derived variables (12-18) in blue and associated nerve and muscle variables (19-21) in green. The 'averages' listed (average human NMJ, average mouse NMJ) for each variable represent the mean for the complete set of NMJs (2860 human; 960 mouse). Fold difference (between human and mouse NMJ) is the ratio of the larger value relative to the smaller value; most NMJ variables (but not all) were larger in mice. Correlation data is listed as coefficient (*r*, numerical) and significance level (*p*, asterisk). [****p< 0.0001, ***p< 0.001, **p< 0.01, *p< 0.05]

Table S2

**Table S2** Patient case series							
case ref	age	sex	side	procedure	indication	DM	function
1R	34	m	R	BKA	non PVD	non DM	mobile
1L	34	m	L	BKA	non PVD	non DM	mobile
2	42	f	L	BKA	non PVD	non DM	mobile
3	49	f	R	BKA	non PVD	non DM	mobile
4	49	m	R	BKA	PVD	DM	mobile
5	50	m	R	BKA	non PVD	non DM	n/a
6	52	f	L	BKA	PVD	DM	n/a
7	56	f	L	BKA	PVD	DM	n/a
8	58	m	L	AKA	PVD	DM	mobile
9	64	m	R	BKA	PVD	DM	n/a
10	66	m	R	BKA	PVD	DM	mobile
11	68	m	R	BKA	PVD	DM	mobile
12	77	m	L	BKA	PVD	DM	n/a
13	79	m	R	BKA	PVD	DM	mobile
14	80	m	R	BKA	PVD	non DM	n/a
15	80	m	R	BKA	PVD	DM	mobile
16	84	m	L	AKA	PVD	DM	mobile
17	85	m	R	AKA	PVD	DM	n/a
18	89	m	L	AKA	PVD	non DM	n/a
19	92	f	L	BKA	PVD	non DM	mobile
20	92	m	L	BKA	PVD	non DM	n/a

Table S2 | Patient case series, Related to Figure 2.

Demographic and clinical details for the complete case series (20 patients). Age is in years. Abbreviations: m, male; f, female; R, right limb; L, left limb; BKA, below knee amputation; AKA, above knee amputation; PVD, peripheral vascular disease; DM, diabetes mellitus; n/a, data not available.

Table S3

Table S3 Human vs mouse muscle metabolic cascades

Ingenuity Canonical Pathways	$-\log_{10}$(*p* value)	Upregulated (H vs M)	Downregulated (H vs M)
Oxidative Phosphorylation	23.90	0/119 (0%)	75/119 (63%)
3-phosphoinositide Degradation	8.24	4/162 (2%)	64/162 (40%)
D-myo-inositol-5-phosphate Metabolism	8.18	4/166 (2%)	65/166 (39%)
D-myo-inositol (1,4,5,6)-tetrakisphosphate Biosynthesis	7.04	4/147 (3%)	57/147 (39%)
D-myo-inositol (3,4,5,6)-tetrakisphosphate Biosynthesis	7.04	4/147 (3%)	57/147 (39%)
tRNA Charging	6.49	2/82 (2%)	35/82 (43%)
3-phosphoinositide Biosynthesis	6.24	6/207 (3%)	69/207 (33%)
Glycolysis I	6.00	0/41 (0%)	22/41 (54%)
Superpathway of Inositol Phosphate Compounds	5.70	6/253 (2%)	79/253 (31%)
Ethanol Degradation II	5.34	1/47 (2%)	24/47 (51%)
Fatty Acid β-oxidation I	5.12	0/45 (0%)	23/45 (51%)
Gluconeogenesis I	4.93	1/46 (2%)	22/46 (48%)
Glutaryl-CoA Degradation	4.77	0/26 (0%)	15/26 (58%)
TCA Cycle II (Eukaryotic)	4.69	0/41 (0%)	20/41 (49%)
Oxidative Ethanol Degradation III	4.46	2/33 (6%)	18/33 (55%)
Ethanol Degradation IV	4.46	1/36 (3%)	19/36 (53%)
Fatty Acid α-oxidation	4.29	1/25 (4%)	15/25 (60%)
Glycogen Degradation III	4.15	0/20 (0%)	12/20 (60%)
Tryptophan Degradation X (Mammalian, via Tryptamine)	4.04	2/35 (6%)	18/35 (51%)
Glycogen Degradation II	3.94	0/18 (0%)	11/18 (61%)
Pyridoxal 5'-phosphate Salvage Pathway	3.67	1/75 (1%)	28/75 (37%)
Valine Degradation I	3.46	0/35 (0%)	16/35 (46%)
Glutathione Redox Reactions I	3.39	1/29 (3%)	14/29 (48%)
Noradrenaline and Adrenaline Degradation	3.24	1/57 (2%)	23/57 (40%)
D-myo-inositol (1,4,5)-trisphosphate Degradation	2.93	0/22 (0%)	11/22 (50%)
Isoleucine Degradation I	2.81	0/29 (0%)	13/29 (45%)
Histamine Degradation	2.70	1/33 (3%)	15/33 (45%)
Putrescine Degradation III	2.42	1/35 (3%)	15/35 (43%)
Superoxide Radicals Degradation	2.27	0/10 (0%)	6/10 (60%)
Salvage Pathways of Pyrimidine Ribonucleotides	2.19	1/112 (1%)	33/112 (29%)
Dopamine Degradation	2.08	1/49 (2%)	18/49 (37%)
Tryptophan Degradation III (Eukaryotic)	2.08	1/49 (2%)	17/49 (35%)
Thioredoxin Pathway	2.05	1/8 (13%)	5/8 (63%)
Glutathione Redox Reactions II	2.05	1/8 (13%)	5/8 (63%)
Calcium Transport I	2.02	0/14 (0%)	8/14 (57%)
Superpathway of Geranylgeranyldiphosphate Biosynthesi	1.97	1/35 (3%)	13/35 (37%)
Ketogenesis	1.88	0/18 (0%)	8/18 (44%)
Ketolysis	1.88	0/18 (0%)	8/18 (44%)
Colanic Acid Building Blocks Biosynthesis	1.86	1/36 (3%)	15/36 (42%)
Glutathione-mediated Detoxification	1.84	0/40 (0%)	15/40 (38%)
Pentose Phosphate Pathway	1.79	1/22 (5%)	9/22 (41%)
Acetyl-CoA Biosynthesis I (Pyruvate Dehydrogenase Comp	1.78	0/12 (0%)	6/12 (50%)
2-ketoglutarate Dehydrogenase Complex	1.77	0/9 (0%)	5/9 (56%)
Branched-chain α-keto acid Dehydrogenase Complex	1.77	0/9 (0%)	5/9 (56%)
Myo-inositol Biosynthesis	1.77	0/9 (0%)	5/9 (56%)
Guanosine Nucleotides Degradation III	1.65	0/23 (0%)	10/23 (43%)
Urate Biosynthesis/Inosine 5'-phosphate Degradation	1.65	0/23 (0%)	10/23 (43%)
Pyrimidine Ribonucleotides Interconversion	1.64	0/54 (0%)	18/54 (33%)
Mevalonate Pathway I	1.61	1/27 (4%)	10/27 (37%)
Stearate Biosynthesis I (Animals)	1.56	1/55 (2%)	18/55 (33%)
Pentose Phosphate Pathway (Oxidative Branch)	1.54	1/10 (10%)	5/10 (50%)
2-oxobutanoate Degradation I	1.50	0/17 (0%)	7/17 (41%)
Adenosine Nucleotides Degradation II	1.50	0/28 (0%)	11/28 (39%)
Folate Transformations I	1.50	1/28 (4%)	11/28 (39%)
Mitochondrial L-carnitine Shuttle Pathway	1.45	0/21 (0%)	9/21 (43%)
Methionine Degradation I (to Homocysteine)	1.41	0/25 (0%)	9/25 (36%)
D-myo-inositol (1,4,5)-Trisphosphate Biosynthesis	1.39	1/37 (3%)	11/37 (30%)
Phosphatidylglycerol Biosynthesis II (Non-plastidic)	1.39	3/41 (7%)	15/41 (37%)
Purine Nucleotides Degradation II (Aerobic)	1.39	0/37 (0%)	13/37 (35%)
Superpathway of D-myo-inositol (1,4,5)-trisphosphate M	1.39	0/33 (0%)	11/33 (33%)
Superpathway of Methionine Degradation	1.38	0/66 (0%)	19/66 (29%)
D-glucuronate Degradation I	1.35	1/11 (9%)	5/11 (45%)
Heme Degradation	1.35	1/11 (9%)	5/11 (45%)
Glucose and Glucose-1-phosphate Degradation	1.33	0/22 (0%)	9/22 (41%)
Pyrimidine Ribonucleotides De Novo Biosynthesis	1.32	0/67 (0%)	20/67 (30%)
Leucine Degradation I	1.31	0/26 (0%)	9/26 (35%)
NADH Repair	1.30	0/8 (0%)	4/8 (50%)
Glycerol-3-phosphate Shuttle	1.30	0/8 (0%)	4/8 (50%)
Hypusine Biosynthesis	1.28	0/5 (0%)	3/5 (60%)
CDP-diacylglycerol Biosynthesis I	1.22	3/35 (9%)	13/35 (37%)
Methylglyoxal Degradation III	1.22	1/23 (4%)	9/23 (39%)
NAD Salvage Pathway II	1.22	1/35 (3%)	12/35 (34%)
Serotonin Degradation	1.21	1/91 (1%)	25/91 (27%)
Methylmalonyl Pathway	1.19	0/12 (0%)	5/12 (42%)
NAD Phosphorylation and Dephosphorylation	1.13	1/20 (5%)	7/20 (35%)
Fatty Acid β-oxidation III (Unsaturated, Odd Number)	1.04	0/6 (0%)	3/6 (50%)
Retinoate Biosynthesis I	1.00	1/38 (3%)	11/38 (29%)
Acetate Conversion to Acetyl-CoA	0.96	0/10 (0%)	4/10 (40%)
Pyruvate Fermentation to Lactate	0.96	0/10 (0%)	4/10 (40%)
Tetrahydrofolate Salvage from 5,10-methenyltetrahydrof	0.96	0/10 (0%)	4/10 (40%)
Trans, trans-farnesyl Diphosphate Biosynthesis	0.96	0/10 (0%)	4/10 (40%)
Acyl-CoA Hydrolysis	0.94	0/14 (0%)	5/14 (36%)
Aspartate Degradation II	0.94	0/14 (0%)	5/14 (36%)
GDP-glucose Biosynthesis	0.93	0/18 (0%)	7/18 (39%)
Choline Degradation I	0.86	0/7 (0%)	3/7 (43%)
GDP-L-fucose Biosynthesis I (from GDP-D-mannose)	0.86	1/7 (14%)	3/7 (43%)
Proline Degradation	0.86	0/7 (0%)	3/7 (43%)
Thiosulfate Disproportionation III (Rhodanese)	0.86	0/7 (0%)	3/7 (43%)
Retinol Biosynthesis	0.86	2/49 (4%)	14/49 (29%)
Chondroitin Sulfate Degradation (Metazoa)	0.85	0/23 (0%)	7/23 (30%)
Heme Biosynthesis II	0.85	0/23 (0%)	7/23 (30%)
Sucrose Degradation V (Mammalian)	0.84	0/19 (0%)	6/19 (32%)
Superpathway of Serine and Glycine Biosynthesis I	0.84	0/19 (0%)	6/19 (32%)
Heme Biosynthesis from Uroporphyrinogen-III I	0.83	0/11 (0%)	4/11 (36%)
Phenylalanine Degradation I (Aerobic)	0.83	0/11 (0%)	4/11 (36%)
Phenylethylamine Degradation I	0.83	0/11 (0%)	4/11 (36%)
Rapoport-Luebering Glycolytic Shunt	0.83	0/11 (0%)	4/11 (36%)
Prostanoid Biosynthesis	0.83	1/15 (7%)	4/15 (27%)
Cysteine Biosynthesis III (mammalia)	0.82	0/32 (0%)	9/32 (28%)
Purine Nucleotides De Novo Biosynthesis II	0.81	0/41 (0%)	11/41 (27%)
Triacylglycerol Degradation	0.74	1/56 (2%)	14/56 (25%)
Superpathway of Citrulline Metabolism	0.73	2/38 (5%)	9/38 (24%)
Ascorbate Recycling (Cytosolic)	0.73	1/12 (8%)	4/12 (33%)
Biotin-carboxyl Carrier Protein Assembly	0.73	1/12 (8%)	3/12 (25%)
Glycogen Biosynthesis II (from UDP-D-Glucose)	0.73	0/12 (0%)	4/12 (33%)
N-acetylglucosamine Degradation II	0.73	0/12 (0%)	4/12 (33%)
NAD Biosynthesis III	0.73	0/12 (0%)	4/12 (33%)
S-methyl-5-thio-α-D-ribose 1-phosphate Degradation	0.73	0/12 (0%)	4/12 (33%)
Formaldehyde Oxidation II (Glutathione-dependent)	0.72	1/8 (13%)	3/8 (38%)
Geranylgeranyldiphosphate Biosynthesis	0.72	0/8 (0%)	3/8 (38%)
N-acetylglucosamine Degradation I	0.72	0/8 (0%)	3/8 (38%)
Retinoate Biosynthesis II	0.72	0/8 (0%)	3/8 (38%)
S-adenosyl-L-methionine Biosynthesis	0.72	0/8 (0%)	3/8 (38%)
Phospholipases	0.66	0/72 (0%)	17/72 (24%)
Triacylglycerol Biosynthesis	0.66	1/58 (2%)	16/58 (28%)
Adenine and Adenosine Salvage III	0.66	0/17 (0%)	5/17 (29%)
Lysine Degradation II	0.66	1/17 (6%)	5/17 (29%)
Citrulline Biosynthesis	0.65	2/26 (8%)	6/26 (23%)
UDP-N-acetyl-D-galactosamine Biosynthesis II	0.65	0/26 (0%)	9/26 (35%)
Arginine Degradation I (Arginase Pathway)	0.63	0/13 (0%)	4/13 (31%)
GDP-mannose Biosynthesis	0.63	0/13 (0%)	5/13 (38%)
Molybdenum Cofactor Biosynthesis	0.63	0/13 (0%)	4/13 (31%)
Pentose Phosphate Pathway (Non-oxidative Branch)	0.63	0/13 (0%)	4/13 (31%)
Adenine and Adenosine Salvage I	0.61	0/9 (0%)	3/9 (33%)
Creatine-phosphate Biosynthesis	0.61	0/9 (0%)	3/9 (33%)
Guanine and Guanosine Salvage I	0.61	0/9 (0%)	3/9 (33%)
The Visual Cycle	0.57	1/32 (3%)	9/32 (28%)
Galactose Degradation I (Leloir Pathway)	0.55	0/14 (0%)	5/14 (36%)
Proline Biosynthesis I	0.55	2/14 (14%)	3/14 (21%)
Serine Biosynthesis	0.55	0/14 (0%)	4/14 (29%)
Fatty Acid Activation	0.52	0/19 (0%)	6/19 (32%)
4-aminobutyrate Degradation I	0.52	0/10 (0%)	3/10 (30%)
Thymine Degradation	0.52	1/10 (10%)	3/10 (30%)
Arginine Biosynthesis IV	0.50	1/24 (4%)	6/24 (25%)
γ-linolenate Biosynthesis II (Animals)	0.50	0/24 (0%)	7/24 (29%)
γ-glutamyl Cycle	0.49	1/29 (3%)	8/29 (28%)
Glycine Biosynthesis I	0.48	0/6 (0%)	2/6 (33%)
S-methyl-5'-thioadenosine Degradation II	0.48	0/6 (0%)	2/6 (33%)
Purine Ribonucleosides Degradation to Ribose-1-phospha	0.47	0/20 (0%)	5/20 (25%)
Glutathione Biosynthesis	0.44	1/11 (9%)	3/11 (27%)
Uracil Degradation II (Reductive)	0.44	1/11 (9%)	3/11 (27%)
Arginine Degradation VI (Arginase 2 Pathway)	0.43	1/16 (6%)	4/16 (25%)
Citrulline-Nitric Oxide Cycle	0.43	1/16 (6%)	4/16 (25%)
Inosine-5'-phosphate Biosynthesis II	0.43	0/16 (0%)	4/16 (25%)
Selenocysteine Biosynthesis II (Archaea and Eukaryotes)	0.43	0/16 (0%)	4/16 (25%)
D-myo-inositol (1,3,4)-trisphosphate Biosynthesis	0.41	0/26 (0%)	6/26 (23%)
Pyrimidine Deoxyribonucleotides De Novo Biosynthesis I	0.40	0/41 (0%)	9/41 (22%)
Aspartate Biosynthesis	0.39	0/7 (0%)	2/7 (29%)
Glutamate Biosynthesis II	0.39	1/7 (14%)	2/7 (29%)
Glutamate Degradation X	0.39	1/7 (14%)	2/7 (29%)
Glutamine Biosynthesis I	0.39	0/7 (0%)	2/7 (29%)
Tyrosine Biosynthesis IV	0.39	0/7 (0%)	2/7 (29%)
UDP-D-xylose and UDP-D-glucuronate Biosynthesis	0.39	0/7 (0%)	2/7 (29%)
Glutamate Degradation III (via 4-aminobutyrate)	0.38	0/12 (0%)	3/12 (25%)
CMP-N-acetylneuraminate Biosynthesis I (Eukaryotes)	0.37	0/17 (0%)	4/17 (24%)
UDP-N-acetyl-D-glucosamine Biosynthesis II	0.37	0/17 (0%)	4/17 (24%)
Choline Biosynthesis III	0.37	0/22 (0%)	5/22 (23%)
Phenylalanine Degradation IV (Mammalian, via Side Chai	0.37	1/32 (3%)	7/32 (22%)
Superpathway of Cholesterol Biosynthesis	0.36	1/83 (1%)	17/83 (20%)
1D-myo-inositol Hexakisphosphate Biosynthesis II (Mamr	0.33	0/28 (0%)	6/28 (21%)
Dermatan Sulfate Degradation (Metazoa)	0.33	0/23 (0%)	5/23 (22%)
Lysine Degradation V	0.33	1/23 (4%)	5/23 (22%)
Folate Polyglutamylation	0.33	0/18 (0%)	4/18 (22%)
Glycerol Degradation I	0.32	0/13 (0%)	3/13 (23%)
Glycine Cleavage Complex	0.32	0/13 (0%)	3/13 (23%)
L-carnitine Biosynthesis	0.32	0/13 (0%)	3/13 (23%)
NAD Salvage Pathway III	0.32	0/13 (0%)	3/13 (23%)
Tetrapyrrole Biosynthesis II	0.32	0/13 (0%)	3/13 (23%)
Alanine Biosynthesis III	0.32	0/3 (0%)	1/3 (33%)
D-mannose Degradation	0.32	0/3 (0%)	1/3 (33%)
Glutamate Removal from Folates	0.32	0/3 (0%)	1/3 (33%)
Lanosterol Biosynthesis	0.32	0/3 (0%)	1/3 (33%)
Acetyl-CoA Biosynthesis III (from Citrate)	0.32	0/8 (0%)	2/8 (25%)
Flavin Biosynthesis IV (Mammalian)	0.32	0/8 (0%)	2/8 (25%)
Methylglyoxal Degradation I	0.32	0/8 (0%)	2/8 (25%)
PRPP Biosynthesis I	0.32	0/8 (0%)	2/8 (25%)
Xanthine and Xanthosine Salvage	0.32	0/8 (0%)	2/8 (25%)
Proline Biosynthesis II (from Arginine)	0.29	1/19 (5%)	4/19 (21%)
dTMP De Novo Biosynthesis	0.28	1/14 (7%)	3/14 (21%)
1,25-dihydroxyvitamin D3 Biosynthesis	0.27	1/9 (11%)	2/9 (22%)
Cardiolipin Biosynthesis II	0.27	0/9 (0%)	2/9 (22%)
Epoxysqualene Biosynthesis	0.27	1/9 (11%)	2/9 (22%)
Glutamate Degradation II	0.27	0/9 (0%)	2/9 (22%)
Glycine Degradation (Creatine Biosynthesis)	0.27	0/9 (0%)	2/9 (22%)
L-cysteine Degradation III	0.27	0/9 (0%)	2/9 (22%)
Methylglyoxal Degradation VI	0.27	0/9 (0%)	2/9 (22%)
Oxidized GTP and dGTP Detoxification	0.27	0/9 (0%)	2/9 (22%)
Urea Cycle	0.25	0/20 (0%)	4/20 (20%)
Acyl Carrier Protein Metabolism	0.24	0/4 (0%)	1/4 (25%)
Sulfite Oxidation IV	0.24	0/4 (0%)	1/4 (25%)
Trehalose Degradation II (Trehalase)	0.22	0/10 (0%)	3/10 (30%)
β-alanine Degradation I	0.22	0/10 (0%)	2/10 (20%)
Adenine and Adenosine Salvage VI	0.18	0/5 (0%)	1/5 (20%)
Anandamide Degradation	0.18	0/5 (0%)	1/5 (20%)
Asparagine Degradation I	0.18	0/5 (0%)	1/5 (20%)
Glutamine Degradation I	0.18	0/5 (0%)	1/5 (20%)
Melatonin Degradation III	0.18	0/5 (0%)	1/5 (20%)
Methylthiopropionate Biosynthesis	0.18	0/5 (0%)	1/5 (20%)
Sorbitol Degradation I	0.18	0/5 (0%)	1/5 (20%)

Table S3 | Conservation of metabolic cascades between human and mouse muscle proteomes, Related to Figure 4.

Out of the 200 metabolic pathways identified in-silico, only 68 show differential proteomic expression between the species (pathways with a $-\log_{10}(p \text{ value}) \geq 1.3$). 66% of the known metabolic cascades are therefore unchanged between human and mouse muscle (NMJ-devoid) samples. Table layout and abbreviations are the same as in Figure 4 (panel d).

Supplemental Experimental Procedures:

Tissue harvesting

Muscle samples were obtained from the amputation specimen in the operating theatre immediately after disconnection of the limb. Tissue was harvested from the proximal end of the specimen, close to the line of surgical incision, in areas demonstrating good back bleeding and muscle fasciculation, and away from areas of necrosis and infection. Any muscle that appeared obviously devitalized on gross inspection was not sampled.

Small blocks of tissue, containing full-length muscle fibres from origin to insertion (approx. 2cm in length) were removed from each of the muscles selected and either immediately fixed in 4% paraformaldehyde for 1 hour or placed on wet ice (depending on the subsequent analyses, see below). All specimens were then transferred from theatre to laboratory for immediate processing.

The major concern relating to the use of tissue harvested from amputation specimens is the potential effect of chronic tissue ischaemia and diabetes mellitus (DM). Although twelve of the twenty patients had one or other form of diabetes, the functional status of all patients (where documented) was commensurate with standing and some degree of walking prior to surgery (with one 92-year-old female able to walk half a mile; Table S2).

At surgery, the level of amputation is principally determined by the likelihood of wound healing; a more distal amputation is the preferred option from a functional point of view, but not at the expense of healing. For the amputation stump to heal

fully the local blood supply must be adequate; thus, tissue sampled close to the line of incision (level of amputation) should also be of adequate health. The final decision on the level of amputation is determined during the procedure itself, by assessing the degree of back bleeding from the stump after disconnection of the limb. If the tissues are clearly devitalized, the amputation is immediately revised to a level at which these conditions are satisfied. These basic principles, along with the frequency with which lower limb amputation is performed (over 4,000 per year in England from 2003-2009) (Ahmad et al., 2014), provided the rationale for utilizing this method of tissue sampling. In addition, all samples were harvested from muscle that demonstrated visible contraction during dissection.

No significant differences were found in mean muscle fibre diameter when comparing patients with and without PVD (Figure 1), excluding any significant sarcopenia and/or disuse atrophy in the present case series. Furthermore, no significant differences were noted in the majority of the core morphological variables measured, when comparing NMJs in patients with or without either PVD (n.s. for 8 of 11 variables) or diabetes (n.s. for 10 of 11) (Figure 1). In addition, the general appearance of NMJs on visual inspection was remarkably conserved even when comparing NMJs at the extremes of the case series (young, non-PVD cf. elderly, PVD; Figure 2).

<u>Muscle dissection and NMJ immunohistochemistry</u>

Following muscle harvest, both human and rodent tissue was processed in an identical manner. Small bundles of 25-30 muscle fibres were teased out from the larger blocks/whole muscles, and of a size suitable for whole-mount preparation. NMJs

were then immunolabelled for presynaptic 2H3/SV2 proteins and postsynaptic AChRs according to the following protocol (antibodies and concentrations are listed below): α-bungarotoxin for 30 minutes; 4% Triton X for 90 minutes; 'block' for 30 minutes (4% bovine serum albumin and 2% Triton X); primary antibodies (in block) for 3 nights at 4°C; 4 washes of 1xPBS; secondary antibodies for 1 night at 4°C; 4 washes of 1xPBS. Preparations were then whole-mounted in Mowiol and stored at -20°C prior to imaging.

Antibodies

Primary antibodies: 1:50 mouse anti-2H3 IgG and 1:50 mouse anti-SV2 IgG (Developmental Studies Hybridoma Bank). Secondary antibodies: 1:50 Alexa Fluor 488 donkey anti-mouse IgG (Thermo Fisher Scientific). 1:500 TRITC α-bungarotoxin (Biotium).

Confocal imaging and NMJ-morph analysis

Images were acquired and analyzed using a standardized workflow, 'NMJ-morph', as previously described (Jones et al., 2016). Briefly, a Zeiss LSM 710 confocal microscope was used to acquire z-stack projections of individual en-face NMJs, including a short length of their pre-terminal axon. Images were then analyzed according to the described workflow, which uses ImageJ or Fiji (and freely availably plugins) to measure 21 individual pre- and post-synaptic morphological variables. Again, based on the NMJ-morph recommendations, 40 NMJs per muscle were analyzed to achieve accuracy of reported mean values. In total, we analyzed 2,860 individual human NMJs and 960 comparative mouse NMJs.

Muscle fibre diameters

Following confocal microscopy, the teased preparations were re-imaged at x20 magnification using an Olympus IX71 microscope, Hamamatsu C4742-95 camera and Openlab Improvision software. Measurement of muscle fibre diameter was performed manually in ImageJ; again, 40 individual fibres were analyzed per muscle (3,820 fibres in total for the complete human/mouse dataset).

dSTORM super-resolution imaging and SNAP25 quantification

A subset of peroneus longus muscle fibres was selected from three human cases. For comparison, an equivalent set of PL muscle fibres were obtained from three CD1 mice. Muscle dissection and immunohistochemistry was performed as described above, with the following modifications: after incubation in the primary antibodies (rabbit anti-SNAP25 IgG, 1:100; Alomone) for 3 nights at 4°C, tissue samples were labeled with Alexa Fluor 647 goat anti-rabbit IgG (1:100; Thermo Fisher Scientific) at room temperature for 2 hours. Specimens were then washed and mounted in dSTORM mounting medium (90% glycerol, 1xPBS, 100mM MEA) (Hou et al., 2015). Human samples underwent an additional 70-minute treatment with 1mM $CuSO_4$ in 50mM ammonium acetate buffer, pH5 (Schnell et al., 1999) to remove lipofuscin-genic autofluorescence after immunolabelling.

Imaging was performed on a customized Nikon Ti Eclipse inverted fluorescent TIRF microscope with a 642nm laser. The lateral stage control was electronic and a piezo focuser (P-725, Physik Instrumente, Germany) precisely controlled the axial position. 30,000-65,000 frames were recorded with a 25ms acquisition time, in a z-step format. Z-steps were repeatedly performed at z-intervals of 200nm. Z-limits were set

manually, slightly above and below the plane of interface between nerve terminal and motor endplate. Only switching events in the given focal plane were accepted by a thresholding filter as part of the PYME implemented jittered triangulation algorithm (Hou et al., 2015). The same settings were used to capture a 550nm LED illuminated widefield stack of TRITC-α-bungarotoxin-labeled AChRs through a Texas Red filter set prior to dSTORM imaging. AChR stacks were used to produce a mean intensity projection image to act as a mask.

Quantification of SNAP25 distribution was performed in an identical manner for all images, using ImageJ. The 'masked' images (SNAP25/AChR overlays) were used as a reference when determining the boundaries of individual boutons/SNAP25 puncta on the dSTORM image, from which all measurements were made. dSTORM images of complete NMJs were first cropped to produce a series of images, each centred on an individual bouton (human) or section of bouton (mouse). The cropped dSTORM images were then converted into 8 bit grayscale counterparts, from which the mean gray value ('intensity') could be measured. These grayscale images were then converted into binary counterparts (using default thresholding) for the subsequent quantification of puncta. Binary images produced by default thresholding provided accurate representations of the original dSTORM images (i.e. no manual adjustments or other thresholding algorithms were required), and this approach was applied consistently for all images. Images were then 'despeckled' to sharpen the edges of individual puncta, and used to quantify the remaining variables: the average area of individual puncta and their density within each bouton, and the total area of all puncta relative to that of each bouton. The derived variable 'total area of active zones per NMJ' (Figure 3) was calculated by multiplying the latter variable (area of puncta :

area of bouton, dSTORM data) by the mean area of synaptic contact for NMJs in peroneus longus (baseline morphological data). Overall, we quantified 50 boutons (n) from 10 NMJs, for 3 individual patients/mice (N) – a total of 2,945 (human) and 10,666 (mice) individual puncta.

Proteomics: tissue sampling

For proteomic analysis, a further subset of PL muscle fibres was selected from four human cases. An equivalent set of PL muscle fibres was obtained from four CD1 mice for comparison. After tissue harvest, muscle dissection was performed on unfixed tissue, in a manner otherwise similar to that described above, to obtain small bundles of 25-30 muscle fibres. In order to identify the location of the endplate bands, muscle fibres were labelled with α-bungarotoxin for 5 minutes; the NMJ-enriched portions of the muscle fibres were then micro-dissected under a Nikon Eclipse 50i fluorescence microscope (Figure 4). NMJ-enriched samples, along with NMJ-devoid muscle 'ends', were then frozen to -80°C prior to proteomic analysis. All muscle dissection and labelling was performed within 30 minutes post-harvest to limit the degree of protein degradation.

Proteomics: protein extraction

Dissected tissue samples were pooled in M tubes (gentle MACS Miltenyi Biotec). Samples were homogenized in label-free extraction buffer [100 mM Tris-HCl (pH7.6) 4% (w/v) SDS] containing 1% protease cocktail inhibitor (Thermo Fisher, UK) using gentle MACS dissociator (Miltenyi Biotec) run on the M tube protein cycle. Post homogenization samples were spun at 300 x g for 2 minutes and left on ice for 20 minutes. Homogenates were transferred to Lo-Bind 1.5ml tubes (Sigma Aldrich) and

centrifuged at 20,000 x g for 20 minutes at 4°C with the soluble fraction of each sample then transferred to new Lo-Bind tubes. Protein determination using the Bicinchoninic acid assay (Pierce, UK) was carried out according to manufacturer's guidelines.

Proteomics: mass spectrometry

Preparation of samples, quantification and bioinformatics was performed according to a standardized protocol (Hughes et al., 2014). Samples were measured out to achieve 200ug/ul protein. DTT was added to give a 10mM final concentration and samples were left on a shaker at room temperature for 30 minutes, followed by incubation with IAA (50mM final concentration) in the dark for 30 minutes. 2ul DTT (1M) was added before samples were frozen.

Hydrophobic and hydrophilic beads (GE HealthCare) were mixed together and washed with MQ water. Beads were transferred to 4 tubes. Samples were then gently added to the beads and mixed/shaken for 2 min. 1% FA was then added to each sample (2 min mix), followed by 50% ACN (8 min mix); samples were then transferred to a magnetic rack and the supernatant was removed. Next, 70% EtOH was added (1 min mix), followed by removal of the supernatant. Finally, ACN was added (1 min mix) and the supernatant again removed.

Proteins were eluted with 100mM TEAB on a shaker for 10 min. Trypsin in 1mM HCl (1ug/ul) was added to each sample and incubated on a shaker at 30°C for 4-6h. A further portion of Trypsin was added and the samples were incubated overnight at 30°C. The tryptic peptides were then labelled with 6-plex TMT reagents (Thermo

Fisher Scientific) using a protocol supplied by the manufacturer [TMT6plex-Nter126 & -Lys126 – Human +, TMT6plex-Nter127 & -Lys127 – Human -, TMT6plex-Nter128 & -Lys128 – Mouse +, TMT6plex-Nter129 & -Lys129 – Mouse -]. The labelled peptides were quenched then mixed together.

The mixed sample was fractionated into 22 fractions using High pH reverse phase chromatography (Ultimate 3000 from Dionex). HPLC buffer A was 10mM ammonium formate in water (pH=10); HPLC buffer B was 10mM ammonium formate in 90% CH_3CN (pH=10). An XBridge peptide BEH column (130Å, 3.5 μm 2.1 X 150 mm from Waters) was used to separate peptides, with the column temperature set to 20°C. Peptides were eluted from the column using a flow rate of 200ul/min and a linear gradient of 5% to 60% buffer B in 60min. 40 fractions were collected and concatenated into 22 fractions based upon UV signal. All fractions were vacuumed dried and re-suspended in 50 μl 1%FA acid prior to MS analysis.

Fractions were analyzed on a Q-exactive-HF mass spectrometer (Thermo Scientific) equipped with Dionex Ultimate 3000 RS and Easyspray column (75 µm x 50 cm, PepMap RSLC C18 column, 2 µm, 100 Å). LC buffer A was 0.1% formic acid in MS grade Milli-Q water; LC buffer B was 80% acetonitrile and 0.08% formic acid in MS grade Milli-Q water. The peptides were eluted from the column using a flow rate of 300nl/min and a linear gradient of 5% to 40% buffer B in 122 min. The column temperature was set to 50°C.

Qexactive HF was performed in data dependent mode: an MS survey scan followed by 15 sequential dependent MS2 scans, with the 15 most intense precursor ions

selected to be fragmented by Higher Energy Collisional Dissociation (HCD), with the isolation window at 0.4 da. The resolution of the MS1 and MS2 was set at 120,000 and 60,000 respectively. The maximum ion injection time for MS1 and MS2 was 50ms and 200ms respectively.

Proteomics: quantification and bioinformatics analysis

The raw mass spectrometric data files obtained for each experiment were collated into a single quantitated dataset using MaxQuant (Cox and Mann, 2008) and Andromeda search engine software (Cox et al., 2011). Enzyme specificity was set to that of trypsin, allowing for cleavage of N-terminal to proline residues and between aspartic acid and proline residues. Other parameters used were: (i) variable modifications, deamidation (NQ), oxidation (M), protein N-acetylation, gln → pyro-glu; (ii) fixed modifications, carbamidomethylation (C); (iii) database: uniprot-reviewed_Mus_A10090_160916 & uniprot-Human-up5640_160516; (iv) Reporter ion MS2 – 4 TMT labels: TMT6plex-Nter126 to 129 & TMT6plex-Lys126 to 129; (v) MS/MS tolerance: FTMS- 10ppm, ITMS- 0.02 Da; (vi) maximum peptide length, 6; (vii) maximum missed cleavages, 2; (viii) maximum of labelled amino acids, 3; and (ix) false discovery rate, 1%. Peptide ratios were calculated using 'Reporter Intensity' for Mouse +/- samples, Human +/- samples and Human/Mouse +/- samples. Data was normalised using 1/median ratio value for each identified protein group per labelled sample. Proteins were filtered to include candidates identified by >1 peptide and demonstrating a >20% change between species. Filtered data was utilised for all bioinformatics statistical analyses including Biolayout Express 3D and Ingenuity Pathway Analyses (IPA). Expression clustering was performed in Biolayout Express 3D software by applying Markov clustering algorithms to raw proteomic data (MCL

2.2). All graphs were clustered using Pearson correlation r=0.96. IPA was performed as previously described (Wishart et al., 2007) with the interaction data limited as follows: direct and indirect interactions; experimentally observed data only; 35 molecules per network; 10 networks per dataset. Prediction activation scores (z-scores) were calculated in IPA.

Proteomics: validation of synaptic enrichment

Human and mouse pooled samples (10 μg load) were separated by SDS-polyacrylamide gel electrophoresis on 4–12% precast NuPage Bis-Tris gradient gels (Life technologies, UK) and then transferred to PVDF membrane using an iBlot 2 fast transfer device (Life Technologies, UK). The membranes were then blocked using Odyssey blocking buffer (Li-cor Biosciences, UK) and incubated with primary antibodies according to the manufacturers' instructions. Secondary antibodies (goat anti-rabbit IRDye 680 and donkey anti-mouse IRDye 680, Li-cor Biosciences, UK) were added according to the manufacturers' instructions. Blots were imaged using an Odyssey Infrared Imaging System (Li-cor Biosciences, UK). Scan resolution of the instrument ranged from 21 to 339 μm, and blots were imaged at 169 μm. Quantification was performed on single channels with the analysis software provided. Total protein stained gels were loaded in parallel with those used for membrane transfer. Gels were stained with InstantBlue (Expedeon) to ensure equal sample loading and were analysed using the Odyssey Infrared Imaging system as previously described (Eaton et al., 2013).

Supplemental References:

Ahmad, N., Thomas, G.N., Gill, P., Chan, C., and Torella, F. (2014). Lower limb amputation in England: prevalence, regional variation and relationship with revascularisation, deprivation and risk factors. A retrospective review of hospital data. J. Royal Soc. Med. *107*, 483-489.

Cox, J., and Mann, M. (2008). MaxQuant enables high peptide identification rates, individualized p.p.b.-range mass accuracies and proteome-wide protein quantification. Nat. Biotechnol. *26*, 1367-1372.

Cox, J., Neuhauser, N., Michalski, A., Scheltema, R.A., Olsen, J.V., and Mann, M. (2011). Andromeda: A Peptide Search Engine Integrated into the MaxQuant Environment. J. Proteome Res. *10*, 1794-1805.

Eaton, S.L., Roche, S.L., Hurtado, M.L., Oldknow, K.J., Farquharson, C., Gillingwater, T.H., and Wishart, T.M. (2013). Total Protein Analysis as a Reliable Loading Control for Quantitative Fluorescent Western Blotting. PLoS One *8*, doi: 10.1371/journal.pone.0072457.

Hou, Y., Jayasinghe, I., Crossman, D.J., Baddeley, D., and Soeller, C. (2015). Nanoscale analysis of ryanodine receptor clusters in dyadic couplings of rat cardiac myocytes. J. Mol. Cell. Cardiol. *80*, 45-55.

Wishart, T.M., Paterson, J.M., Short, D.M., Meredith, S., Robertson, K.A., Sutherland, C., Cousin, M.A., Dutia, M.B., and Gillingwater, T.H. (2007). Differential proteomics

analysis of synaptic proteins identifies potential cellular targets and protein mediators of synaptic neuroprotection conferred by the slow Wallerian degeneration (Wld^{s}) gene. Mol. Cell. Proteomics *6*, 1318-1330.
