# Supplementary material for: Cellular and Molecular Anatomy of the Human Neuromuscular Junction
Source: Cell Rep. 2017 Nov 28;21(9):2348–56. doi: 10.1016/j.celrep.2017.11.008 (PMC5723673; doi:10.1016/j.celrep.2017.11.008)
Supplement: Document S2. Article plus Supplemental Information [file mmc2.pdf]

Cell Reports

Report

Cellular and Molecular Anatomy of the Human Neuromuscular Junction

Graphical Abstract

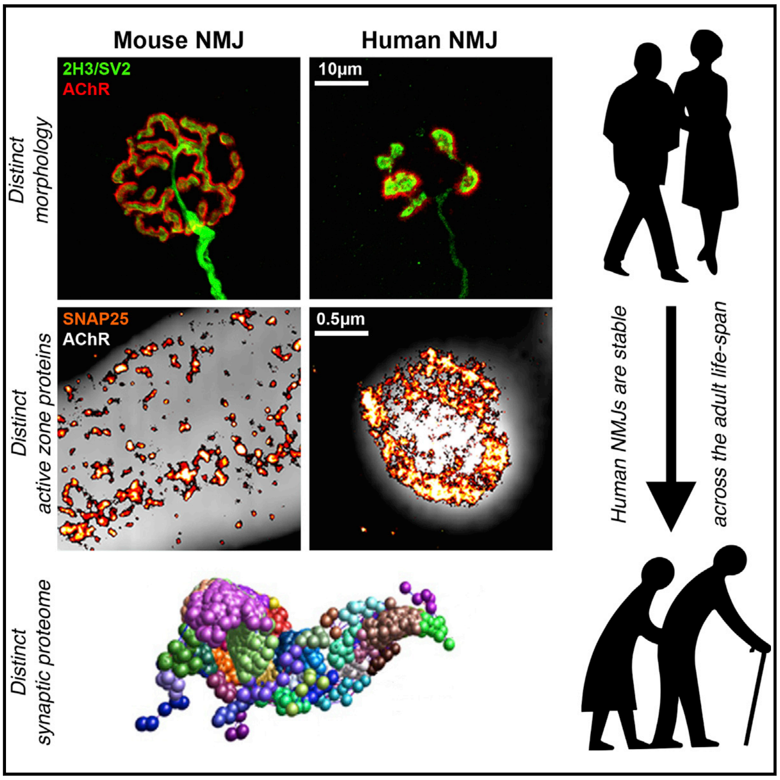

Authors

Ross A. Jones, Carl Harrison, Samantha L. Eaton, ..., Christian Soeller, Thomas M. Wishart, Thomas H. Gillingwater

Correspondence

t.gillingwater@ed.ac.uk

In Brief

Jones et al. reveal fundamental differences between synapses in humans and lower mammals. They show that human neuromuscular junctions (NMJs) are smaller and more fragmented than comparable synapses from mice, with a distinct molecular composition. In contrast to mice, human NMJs were also remarkably stable across the entire adult lifespan.

Highlights

- Human neuromuscular junctions (NMJs) are morphologically distinct from rodent NMJs
- Human NMJs are remarkably stable across the adult lifespan
- Active zone proteins, including SNAP25, are differentially localized in human NMJs
- Significant divergence between the synaptic proteome of human and mouse NMJs

Jones et al., 2017, Cell Reports *21*, 2348–2356
November 28, 2017 © 2017 The Author(s).
https://doi.org/10.1016/j.celrep.2017.11.008

OPEN ACCESS
CellPress

Cell Reports
Report

Cellular and Molecular Anatomy of the Human Neuromuscular Junction

Ross A. Jones,[1,2] Carl Harrison,[3] Samantha L. Eaton,[4] Maica Llavero Hurtado,[4] Laura C. Graham,[4] Leena Alkhammash,[1] Oladayo A. Oladiran,[1] Andy Gale,[1] Douglas J. Lamont,[5] Hamish Simpson,[6] Martin W. Simmen,[1] Christian Soeller,[3] Thomas M. Wishart,[2,4] and Thomas H. Gillingwater[1,2,7,*]
[1]Edinburgh Medical School: Biomedical Sciences, University of Edinburgh, Edinburgh EH8 9AG, UK
[2]Euan MacDonald Centre for Motor Neurone Disease Research, University of Edinburgh, Edinburgh EH8 9AG, UK
[3]Physics and Astronomy, University of Exeter, Exeter EX4 4QL, UK
[4]Neurobiology, Roslin Institute, University of Edinburgh, Edinburgh EH25 9RG, UK
[5]Fingerprints Proteomics, University of Dundee, Dundee DD1 5EH, UK
[6]Department of Orthopaedic Surgery, University of Edinburgh, Edinburgh EH16 4SB, UK
[7]Lead Contact
*Correspondence: t.gillingwater@ed.ac.uk
https://doi.org/10.1016/j.celrep.2017.11.008

SUMMARY

The neuromuscular junction (NMJ) plays a fundamental role in transferring information from lower motor neuron to skeletal muscle to generate movement. It is also an experimentally accessible model synapse routinely studied in animal models to explore fundamental aspects of synaptic form and function. Here, we combined morphological techniques, super-resolution imaging, and proteomic profiling to reveal the detailed cellular and molecular architecture of the human NMJ. Human NMJs were significantly smaller, less complex, and more fragmented than mouse NMJs. In contrast to mice, human NMJs were also remarkably stable across the entire adult lifespan, showing no signs of age-related degeneration or remodeling. Super-resolution imaging and proteomic profiling revealed distinctive distribution of active zone proteins and differential expression of core synaptic proteins and molecular pathways at the human NMJ. Taken together, these findings reveal human-specific cellular and molecular features of the NMJ that distinguish them from comparable synapses in other mammalian species.

INTRODUCTION

Synapses play fundamental roles in the form and function of the nervous system both in health and during disease. Despite numerous important breakthroughs in our understanding of the cellular and molecular composition of synapses in animal models, both historic (Fatt and Katz, 1952; Couteaux and Pécot-Dechavassine, 1970; Nitkin et al., 1987) and recent (Oh et al., 2014; Wilhelm et al., 2014; Kasthuri et al., 2015), we know surprisingly little about the equivalent make up of synapses in humans. Current studies of synaptic connectivity at the cellular and molecular level have therefore relied heavily on "model" organisms, both vertebrate and invertebrate, working on the tacit assumption that the biological principles uncovered can ultimately be applied to humans.

The neuromuscular junction (NMJ) represents one major subclass of synapse in the mammalian nervous system, critical for the transfer of information between the nervous system (lower motor neuron) and skeletal muscle. It also epitomizes a "model" synapse (Shi et al., 2012), both conveniently accessible within the peripheral nervous system and an early target in several neurodegenerative conditions, including amyotrophic lateral sclerosis and spinal muscular atrophy (Murray et al., 2010). Indeed, many of the fundamental principles governing synaptic form and function in the nervous system were discovered from early experiments examining NMJs in model organisms (Slater, 2015). More recently, the NMJ has been used to reveal core aspects of synaptic form and function *in vivo*, including the control of activity-dependent plasticity (Newman et al., 2017), as well as synaptic development and age-related decline (Liu et al., 2017).

Surprisingly, however, compared to extensive experimental data from animal models, there is currently a relative paucity of data concerning the cellular and molecular composition of the human NMJ. Ethical considerations and the logistics of obtaining biopsy material (in contrast to post-mortem sampling) from healthy individuals during life make it difficult to obtain tissue samples that are suitably well-preserved to facilitate high-resolution cellular and molecular analysis (Kay et al., 2013). Here, we report the development of a tissue harvesting and processing approach during surgical amputation that has allowed us to undertake a detailed cellular and molecular characterization of the healthy human NMJ across the adult lifespan.

RESULTS AND DISCUSSION

Tissue samples were obtained from surgical discard material from twenty patients undergoing lower limb amputation for a variety of clinical indications (for full patient details, see Experimental Procedures; summarized in Table S2), including complications of peripheral vascular disease (PVD) and non-PVD-related cases (e.g., for chronic pain following previous orthopedic surgery or chronic osteomyelitis refractory to antibiotic treatment). Importantly, samples were obtained from

 © 2017 The Author(s).
This is an open access article under the CC BY license (http://creativecommons.org/licenses/by/4.0/).

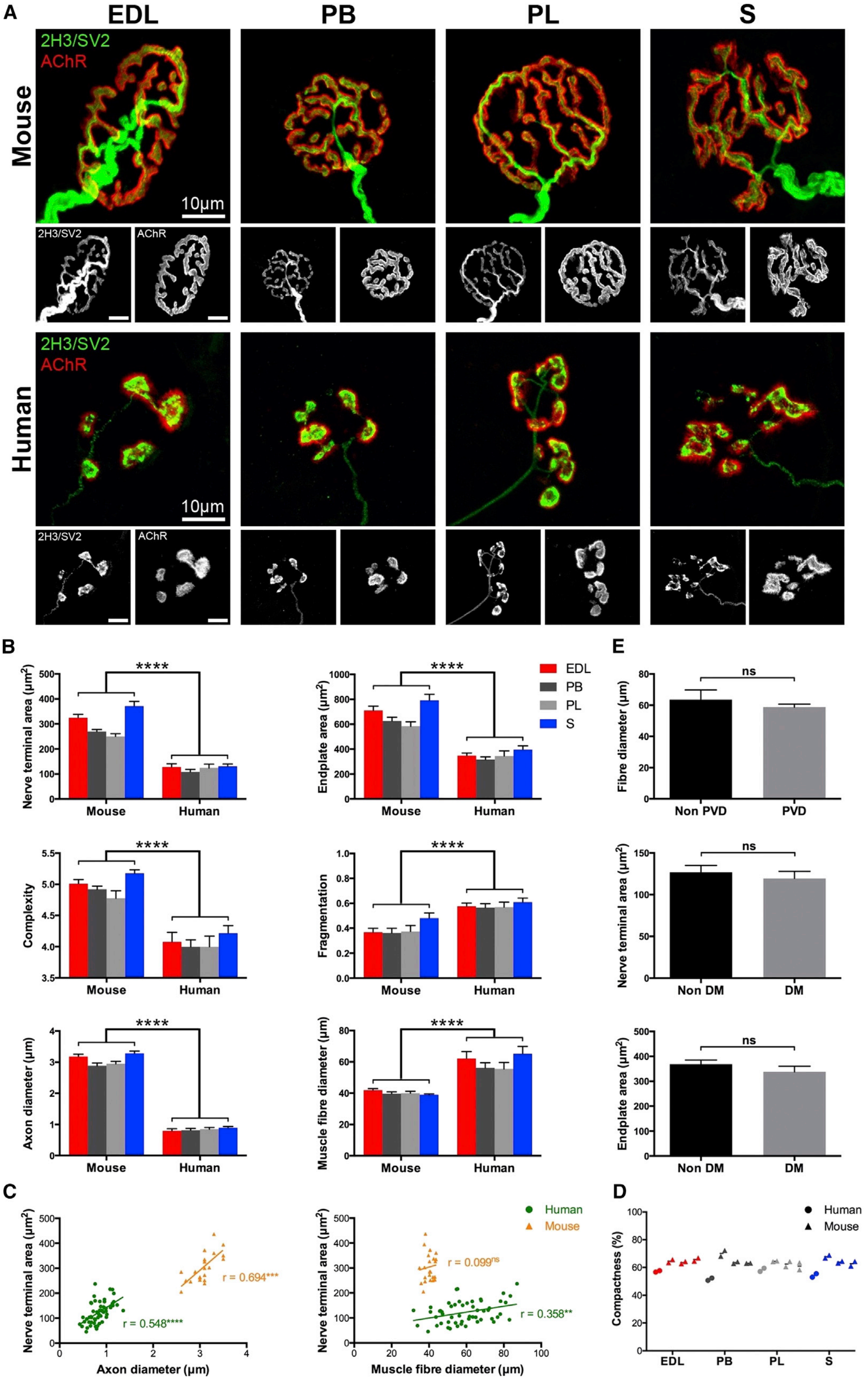

(legend on next page)

non-pathological—otherwise healthy—regions of limb (e.g., close to the site of amputation) where the tissue needs to be free from any disease or pathology in order for sufficient post-operative tissue healing to occur. NMJs from four anatomically distinct muscles were harvested: *extensor digitorum longus* (EDL), *soleus* (S), *peroneus longus* (PL), and *peroneus brevis* (PB). For comparison, the same muscles were dissected from a single litter of young adult CD1 mice. NMJs were immunohistochemically labeled, imaged, and analyzed using a standardized platform: "NMJ-morph" (Jones et al., 2016). For each NMJ, 21 individual synaptic variables were measured. Baseline data were obtained for 2,860 human NMJs across seven decades of life (from the ages of 34–92 years).

Cellular Architecture of the Human NMJ

Initial qualitative observations revealed striking morphological differences between human and mouse NMJs (Figure 1). Human NMJs were universally smaller than their mouse counterparts, with much thinner pre-terminal axons, more rudimentary nerve terminals, and distinctive "nummular" (formed of coin-shaped patches) endplates (Figure 1). These observations were confirmed quantitatively (Table S1). Axon diameter and average area of AChR clusters showed the greatest differences between species (3.69-fold**** and 3.33-fold****, respectively; higher in mice), with over half of the morphological variables studied (12 of 21) showing a fold difference of at least 150% between humans and mice. The degree of overlap between pre- and post-synaptic components (the proportion of AChR labeling at the motor endplate that is directly covered by overlying motor nerve terminal) was relatively similar between the species (50% in humans, 64% in mice), in agreement with previous electron microscopy (EM) studies (Slater et al., 1992). None of the human NMJs analyzed lacked pre-synaptic boutons overlying AChR clusters (Figure 1); the only difference was the degree to which AChRs were dispersed beyond the limits of the nerve terminals. Thus, each and every individual AChR island at the motor endplate was innervated by a corresponding motor nerve terminal bouton. Importantly, no significant morphological differences at the human NMJ could be attributed to patient co-morbidities (diabetes mellitus, vascular disease) (Figure 1; also see Experimental Procedures), and the side of the body examined did not influence morphology (Figure 1), consistent with our previous findings in mice (Jones et al., 2016).

Although human NMJs were routinely only half the size of their mouse counterparts, with axons only a third of the caliber, they were found to innervate muscle fibers up to twice the diameter of those in mice (Figure 1). We therefore assessed the relationship between NMJ morphology and pre- and post-synaptic cells by correlating each morphological variable with motor axon diameter and muscle fiber diameter respectively. In both species, NMJ morphology correlated more strongly with motor axon diameter (Jones et al., 2016) (Figure 1; Table S1). Correlation coefficients (r) were higher in relation to axon diameter for the majority of NMJ variables in both species (10 of the 18), with only a minority (6 in humans, 1 in mice) being more closely correlated with the muscle fiber, suggesting that the morphological properties of the motor neuron (as determined by measuring axon diameter) exerted a stronger influence on synaptic morphology in humans than morphological properties of the skeletal muscle fiber.

To confirm that the differences in NMJ morphology we observed between humans and mice were a consistent observation between humans and other mammals, we also compared our human and mouse data with NMJs from adult rats (Figure S1). Here, NMJ morphology was virtually indistinguishable between mice and rats across all muscles examined, with both species being clearly distinctive from humans. In addition, this comparison allowed us to establish whether the relatively small size of human NMJs is simply a consequence of increased body size. As an adult rat is considerably larger than a comparable mouse (~10-fold increase in body weight), if NMJ size was inversely correlated with body size, we would have expected rat NMJs to be notably smaller than mouse NMJs (e.g., an intermediate NMJ size, somewhere between mice and humans). As NMJs from mice and rats were morphologically indistinguishable from each other, we conclude that the smaller size of human NMJs cannot simply be a consequence of increased body size and mass (Figure S1).

Taken together, these findings reveal that human NMJs have a unique morphology, being significantly smaller and more fragmented than comparable synapses from widely used animal models (mice and rats). This challenges the simplistic assumption that structures in the human nervous system are inevitably larger and more complex than those in lower mammals.

The Human NMJ across the Lifespan

One area of research that is currently receiving significant interest concerns an apparent age-related decline in synaptic stability at the NMJ, manifesting as degenerative changes affecting both the pre-synaptic motor nerve terminal and the

Figure 1. Unique Morphology of the Human NMJ

(A) Representative confocal micrographs from 4 lower limb muscles. Human NMJs are smaller, with a thinner axon, less complex nerve terminals and a distinctive "nummular" endplate. Scale bar, 10 μm.

(B) Bar charts demonstrating significant species-specific differences across a range of pre- and post-synaptic variables. Each bar represents the mean (±SEM) of >600 human NMJs (and 240 mouse NMJs). Unpaired t test and Mann-Whitney test.

(C) Scatterplots showing correlation between nerve terminal area and axon/muscle fiber diameter. Each data point is an individual muscle (mean of 40 NMJs) (72 human and 24 mouse muscles). NMJ morphology was more closely correlated with structural features of the pre-synaptic cell (motor axon) in both species. Pearson correlation.

(D) Left/right muscles pairs from the same human case were compared with 3 mouse controls. Each data point (in the pair) is an individual muscle (left or right); the intervening line is the mean of the 2 sides. Laterality did not influence NMJ morphology in either species.

(E). Effect of co-morbidities on human NMJ morphology. No significant morphological differences could be attributed to either diabetes mellitus (DM) or peripheral vascular disease (PVD). Unpaired t tests.

****$p < 0.0001$, ***$p < 0.001$, **$p < 0.01$, *$p < 0.05$.

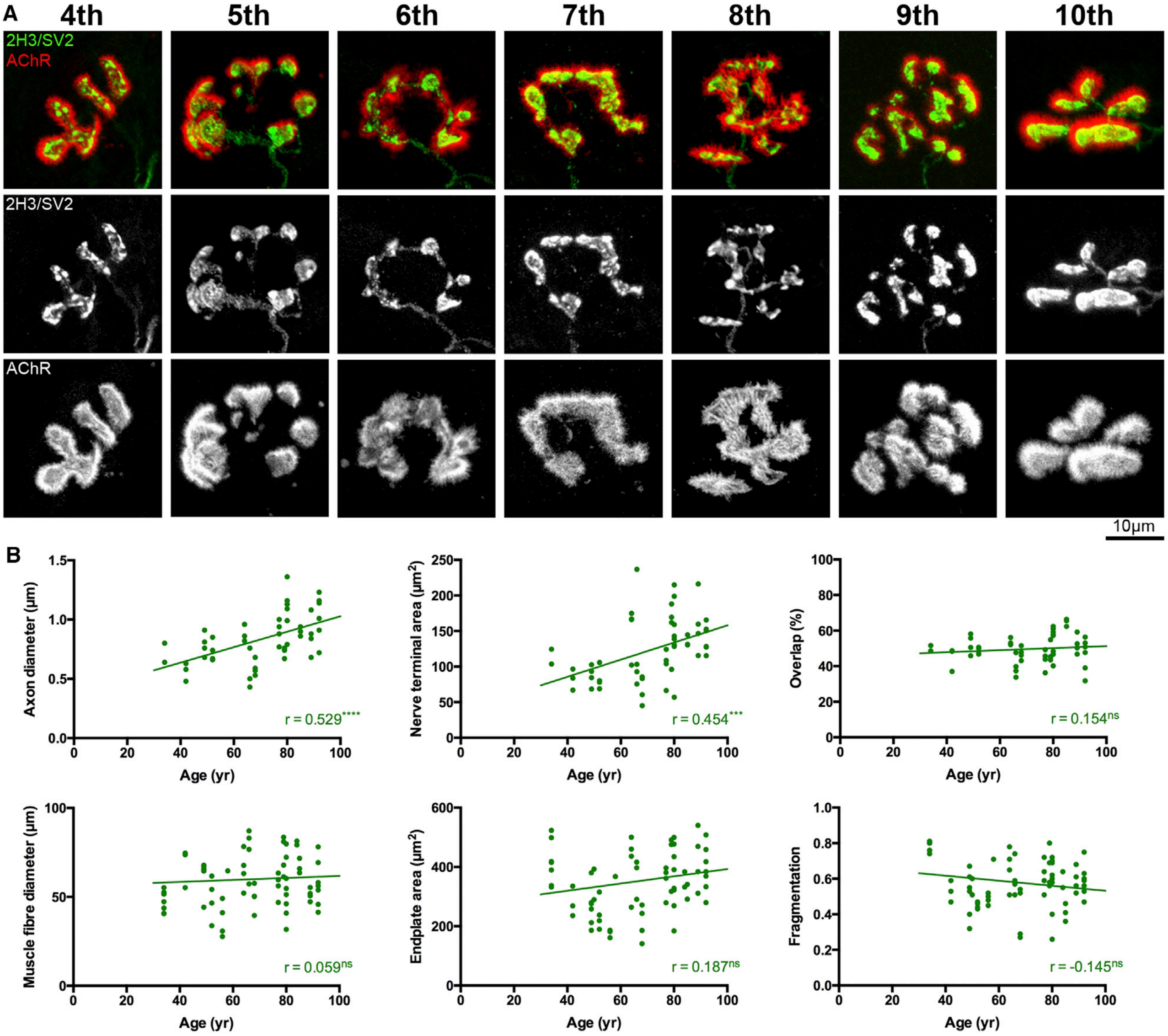

Figure 2. The Human NMJ Is Stable across the Lifespan

(A) Representative confocal micrographs of human NMJs from the 4th to the 10th decades of life (all from *peroneus longus* muscle). Despite the heterogeneity of individual NMJs, the overall appearance is conserved across the 70+ year age range. Scale bar, 10 μm.

(B) Scatterplots showing correlations between age and a range of individual pre- and post-synaptic NMJ variables. Data are pooled across the 4 muscle groups (72 individual muscles). Each data point is an individual muscle (mean of 40 NMJs). Although 2 of the pre-synaptic variables shown correlated with age (a modest increase in the size of the pre-synaptic axon and motor nerve terminal), overall synaptic morphology remained remarkably stable. Pearson and Spearman correlation.

****$p < 0.0001$, ***$p < 0.001$.

post-synaptic motor endplate (Gonzalez-Freire et al., 2014; Liu et al., 2017). Although findings from animal models (Anis and Robbins, 1987; Balice-Gordon and Lichtman, 1990; Valdez et al., 2010; Willadt et al., 2016) suggest that NMJs are inherently unstable with age, it is unclear whether a similar phenomenon occurs across the longer human lifespan. We were able to address this important question as our human tissue samples incorporated patients from the fourth to the tenth decades of life, with the individual ages of patients distributed approximately evenly across the age range (Table S2).

Qualitative analyses of NMJs suggested conservation of synaptic structure across the entire lifespan in humans (Figure 2). The only change observed with increasing age was a modest increase in axon diameter (r = 0.529****) (Figure 2). Notably, there was no significant change in muscle fiber diameter or endplate area with age. Nor were there any age-associated changes in the degree of overlap between pre- and post-synaptic components of the NMJ, or any evidence for age-associated fragmentation of the NMJ (Figure 2). Furthermore, the modest degree of pre-synaptic remodeling that occurred with age was de-coupled

from equivalent changes in the muscle fiber, further supporting our finding that synaptic morphology is more closely correlated with the pre-synaptic neuron. Thus, the human NMJ remains remarkably stable across the adult lifespan, devoid of any of the age-related degeneration and/or remodeling changes that have been reported in other mammalian species occurring over a much shorter time scale (Valdez et al., 2010; Willadt et al., 2016).

Interestingly, these findings partially contradict an earlier study suggesting that the human NMJ undergoes changes over the adult lifespan (Oda, 1984). These differences are most likely explained by methodological disparities between the studies and a smaller sample size in the Oda (1984) study. For example, Oda used relatively low-resolution techniques (e.g., silver and cholinesterase staining) to study 12 autopsy samples obtained 6 hr after death. Our own preliminary experiments confirmed that we needed to harvest tissue quickly (e.g., within minutes) from freshly biopsied material (not post-mortem) in order to obtain accurate morphological measurements. Moreover, our findings are in agreement with another smaller study of human muscle samples where endplate size was found to remain stable with age (Wokke et al., 1990).

Super-Resolution (Direct Stochastic Optical Reconstruction Microscopy) Imaging of Active Zone Proteins at the Mouse and Human NMJ

Given the structural differences we observed between human and mouse NMJs, we next wanted to establish whether human NMJs were also distinct from the molecular perspective. In our initial morphological experiments, labeling of the synaptic vesicle protein SV2 appeared to be qualitatively different between human and mouse nerve terminals. Synaptic boutons in mice were characterized by relative homogeneity of labeling, whereas motor nerve terminals at human NMJs contained distinctive "hotspots" of fluorescence (particularly clear examples can be seen in the 2H3/SV2 greyscale panels for the 4th, 7th, and 9th decade images shown in Figure 2). Because the functional architecture of active zones varies considerably between species (Ackermann et al., 2015), we hypothesized that the heterogeneous labeling of SV2 in humans reflected differential spatial arrangement of active zone proteins/material at human synapses.

We assessed active zone protein distribution in human NMJs by performing direct stochastic optical reconstruction microscopy (dSTORM) super-resolution imaging (Huang et al., 2010) of one key active zone protein (SNAP25). SNAP25 is a component of the SNARE complex, a series of structural proteins responsible for the fusion of synaptic vesicles with the plasma membrane during exocytosis (Han et al., 2017), that is also known to be localized to active zones *in vivo* (Wilhelm et al., 2014). dSTORM imaging at the mouse NMJ revealed a remarkably similar punctate distribution of SNAP25 to that previously reported from studies of other active zone proteins in a variety of species using a range of different techniques (Fukunaga et al., 1983; Meinertzhagen et al., 1998; Nagwaney et al., 2009). For example, quantitative comparisons between our mouse SNAP25 data and recent data from STED microscopy experiments studying the active zone proteins Bassoon and Piccolo in mice (Nishimune et al., 2016) revealed remarkably similar subcellular distribution: the average density of Bassoon and Piccolo "puncta" in the Nishimune et al. (2016) study was $\sim$10 per μm^2, with similar analyses of SNAP25 in our study revealing a density of $\sim$15 per μm^2.

The finding that dSTORM imaging could be used to reliably label and quantify SNAP25 distribution at the mouse NMJ prompted us to apply dSTORM imaging to compare SNAP25 localization between mouse and human NMJs. Parallel dSTORM imaging of human and mouse NMJs revealed clear differences in the distribution and intensity of SNAP25 between the two species (Figure 3). We quantified a total of 2,945 (human) and 10,666 (mouse) individual SNAP25 puncta, from 50 boutons (10 NMJs) of 3 individual patients/mice. All four core variables measured were found to be significantly greater in humans than in mice: the average area of individual SNAP25 puncta and their density within each bouton (both $^{**}p < 0.01$), the total area of all puncta relative to that of each bouton ($^{****}p < 0.0001$), and the intensity of SNAP25 labeling ($^{****}p < 0.0001$).

Given that motor nerve terminals (and hence the "area of synaptic contact") at the human NMJ are significantly smaller than those in mice (Figure 1; Table S1), we calculated the total area of SNAP25 labeling per NMJ. This analysis revealed the total area to be identical in both humans and mice: approximately 15 μm^2 of SNAP25 per NMJ (Figure 3). Therefore, although the human NMJ is significantly smaller than the mouse NMJ, the total amount of SNAP25 protein (and, by extension, the amount of molecular active zone machinery) is similar between human and mouse NMJs, albeit packaged into significantly smaller synaptic boutons in humans. This observation suggests the possible presence of a homeostatic mechanism that preserves the functional architecture of the synapse in the face of significant morphological heterogeneity. In conjunction with the extensive post-synaptic junctional folding demonstrated previously (Slater et al., 1992), this pre-synaptic specialization could play a role in effectively maintaining neurotransmission at the smaller human NMJ. The application of super-resolution imaging to visualize active zone proteins at the human NMJ reported here paves the way for future studies that will be able to develop a more refined and detailed subcellular "map" of synaptic protein distribution and localization across different species. Such studies may also assist in addressing the apparent discrepancies that exist between the species-specific differences we report with respect to the molecular composition of active zones and previous freeze-fracture studies that suggested more consistent conservation of gross active zone structure between human and rodent synapses (for review see Rogers and Nishimune, 2017). Of note, we were unable to reliably label human NMJs with antibodies against Bassoon or Piccolo, which may point to species-specific differences in these antigens.

Molecular Profiling of the Human NMJ

To investigate potential differences in the broader species-specific molecular composition of the human NMJ, we next utilized state-of-the-art proteomic techniques (tandem mass tagging) to undertake comprehensive proteome-wide profiling of human and mouse NMJs. By establishing the proteomic profile of micro-dissected NMJ-enriched and NMJ-devoid skeletal muscle

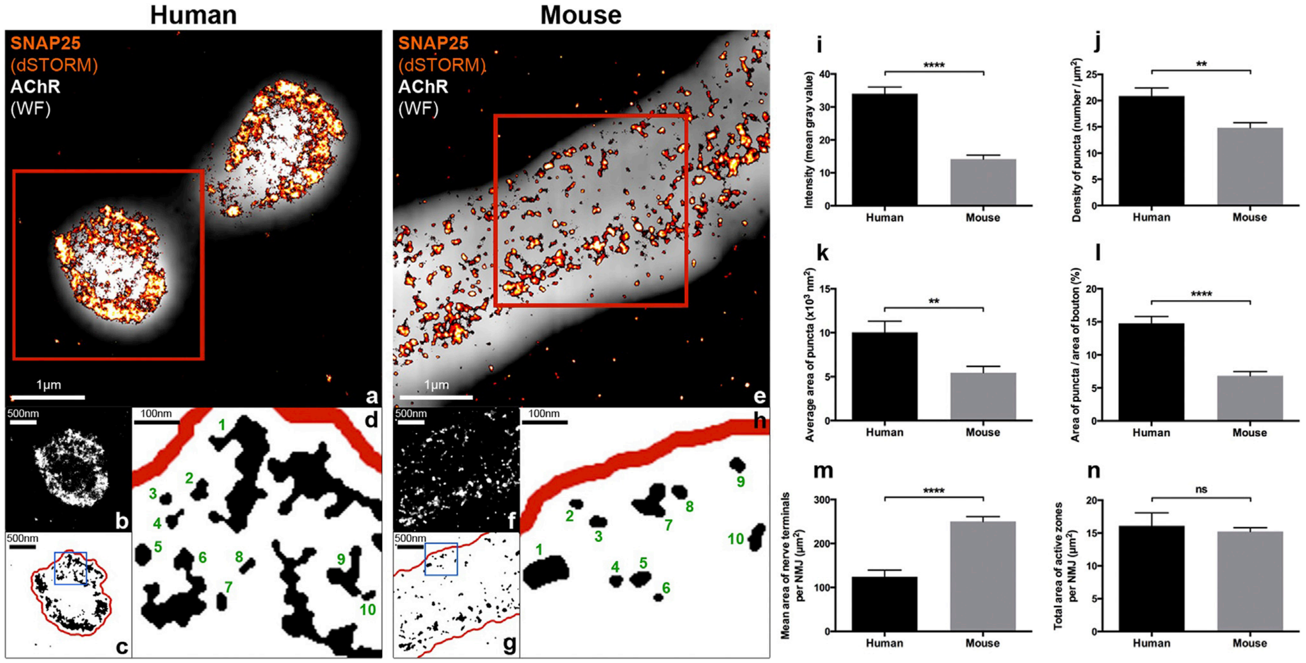

Figure 3. Comparative Super-Resolution (dSTORM) Imaging of the Active Zone Protein SNAP25 at Human and Mouse NMJs

(A and E) Composite images of SNAP25-labeled nerve terminals (dSTORM, orange/red) overlaid on BTX-labeled AChRs of the motor endplate (wide-field, gray). Note that (A) only shows two single synaptic boutons from the human NMJ (not the whole NMJ), and (B) only shows a sub-region of one single synaptic bouton from the mouse NMJ (again not the whole NMJ). Scale bars, 1 μm.

(B and F) 8-bit greyscale images of single boutons (representing the areas contained within the red boxes in A and E) used to quantify intensity of labeling (I). Note the increased intensity and density of SNAP25 in the human NMJ. Scale bars, 500 nm.

(C and G) Despeckled, binary versions of (B) and (F) used to quantify the remaining variables (J–L). The boxed areas have been enlarged (D and H) to depict individual SNAP25 puncta; 10 discrete puncta have been labeled in each image. Scale bars, 100 nm.

(I–L) Bar charts showing dSTORM image quantification of SNAP25 intensity (I), density of SNAP25 puncta (J), average area of SNAP25 puncta (K), and the area of SNAP25 puncta as a percentage of total bouton area (L), at human and mouse NMJs. All 4 measures of SNAP25 labeling were significantly greater in the human NMJs. For both human and mouse datasets, n = 50 boutons; bar charts depict mean (±SEM). Unpaired t test and Mann-Whitney test.

(M and N) Bar charts comparing the average size of human and mouse NMJs (nerve terminal area, M) with the total size of their active zone material (SNAP25 area per NMJ, N). Although the human NMJ is significantly smaller than the mouse NMJ (M), the total amount of SNAP25 labeling at the NMJ is the same when adjusted to reflect the total overall size of the synapse (N). Unpaired t test.

****$p < 0.0001$, **$p < 0.01$.

samples (Figures 4 and S2), we were able to establish and compare the protein-level composition of NMJs and muscle fibers in human and mouse samples. Through a combined human/mouse database search we confirmed the identification of peptides associated with 6,737 proteins. Application of stringent filtering parameters in order to ensure reliability of protein identification subsequently yielded 5,026 proteins for bioinformatics analyses. This represents a high level of coverage from a single proteomic analysis and compares favorably with a recent database of human muscle proteins identified by mass spectrometry that lists 5,431 muscle proteins across 38 peer reviewed scientific publications from 2002–2017 (Gonzalez-Freire et al., 2017). Moreover, we were able to demonstrate the identification of synaptic and neuronal proteins within our NMJ-enriched dissections (Figures S3 and S4).

Comparative bioinformatics analysis of proteomic profiles from human and mouse skeletal muscle samples (devoid of NMJs) revealed clear molecular overlap (Figure 4; Table S3), with the majority (66%) of the 200 known metabolic cascades identified showing no significant species-dependent differences. In contrast, the expression profiles for the NMJ-enriched samples indicated a clear molecular variation between humans and mice (Figure 4). For example, *in silico* analysis identified 36 distinct nervous system-related molecular pathways known to impact on NMJ form and function (including a range of core synaptic signaling pathways, such as "integrin signaling," "axonal guidance signaling," and "CREB signaling in neurons") where proteins were present in both human and mouse datasets. Surprisingly, 97% of these pathways showed statistically significant differences in protein expression levels between the two species (Figures 4 and S4). For example, major differences were observed in the levels of 24 individual proteins contributing to agrin signaling pathways at the NMJ (Figure S4). Importantly, each affected pathway included some proteins that were more abundant in human samples as well as other proteins that were more abundant in mouse samples, confirming that the differences observed were not simply an artifact of relative enrichment during tissue processing. Thus, these proteomics datasets provide evidence to suggest that human NMJs have a significantly modified molecular composition compared to analogous

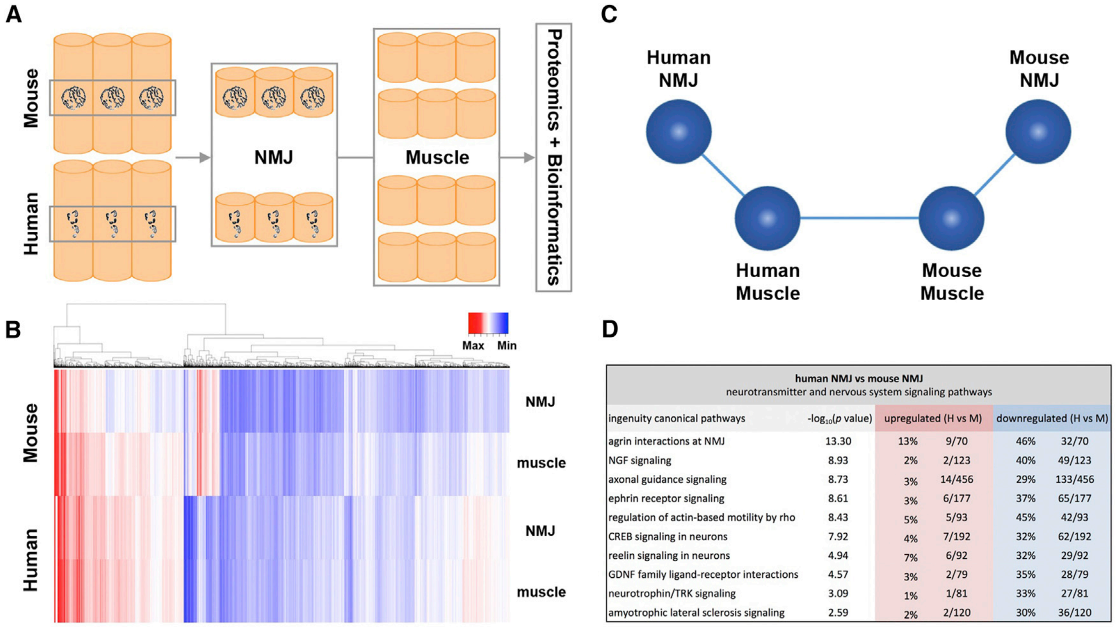

human NMJ vs mouse NMJ neurotransmitter and nervous system signaling pathways					
ingenuity canonical pathways	$-\log_{10}(p$ value)	upregulated (H vs M)		downregulated (H vs M)	
agrin interactions at NMJ	13.30	13%	9/70	46%	32/70
NGF signaling	8.93	2%	2/123	40%	49/123
axonal guidance signaling	8.73	3%	14/456	29%	133/456
ephrin receptor signaling	8.61	3%	6/177	37%	65/177
regulation of actin-based motility by rho	8.43	5%	5/93	45%	42/93
CREB signaling in neurons	7.92	4%	7/192	32%	62/192
reelin signaling in neurons	4.94	7%	6/92	32%	29/92
GDNF family ligand-receptor interactions	4.57	3%	2/79	35%	28/79
neurotrophin/TRK signaling	3.09	1%	1/81	33%	27/81
amyotrophic lateral sclerosis signaling	2.59	2%	2/120	30%	36/120

Figure 4. Unique Molecular Profile of the Human NMJ

(A) Schematic representation of sample micro-dissection used to produce NMJ-enriched and NMJ-devoid (muscle) samples for proteomics and subsequent bioinformatics analysis. NMJ presence/absence was confirmed by α-bungarotoxin labeling (Experimental Procedures).

(B) Heatmap representation of proteomic data. Abundance is indicated by color intensity, from lowest (darkest blue) to highest (darkest red). Clear differences exist between the 4 samples, particularly when comparing human and mouse NMJs.

(C) Schematic representation of proteomic data based on Biolayout output (Experimental Procedures). Each sphere represents the entire expression data for an individual tissue sample, and the proximity and orientation relative to one another indicates the similarity of the datasets; human and mouse muscle samples are more similar than human and mouse NMJ samples.

(D) *In silico* analysis identifies canonical cascades relating to neurotransmitter function and nervous system signaling. Ten example pathways are listed. Values are the percentage (and number) of proteins in each cascade that are upregulated (red) or downregulated (blue) in human cf. mouse. Significance levels are listed as $-\log_{10}$(p value), i.e., $-\log_{10}(p < 0.05) \approx 1.3$, $-\log_{10}(p < 0.0001) = 4$, etc. Of the 36 pathways identified, only 1 showed no significant species difference (GABA receptor signaling cascade; not shown).

NMJs in mice. This finding is consistent with recent observations of species-specific gene expression in developing neurons from humans and mice (Qiu et al., 2016).

Taken together, our findings reveal human-specific features of the NMJ that distinguish them from comparable synapses in other mammalian species. These fundamental differences between synapses in humans and lower mammals must be taken into careful consideration when interpreting animal-based studies with respect to their applicability to humans.

EXPERIMENTAL PROCEDURES

Further details and an outline of resources used in this work can be found in Supplemental Experimental Procedures.

Ethics

Use of anonymous human tissue was granted by the Lothian NRS BioResource (SR719, 15/ES/0094); prospective tissue collection was approved by the Lothian Ethics Committee (REC 2002/1/22, 2002/R/OST/02) following internal (University of Edinburgh) and independent/external ethical review.

Human Case Series

Human muscle samples were obtained from patients following lower limb amputation surgery (see above for ethical/institutional approvals). In total, 21 sets of muscle samples were obtained from 20 patients (15 male, 5 female)—1 patient required a bilateral procedure. The clinical details for each patient are summarized in Table S2. The majority of patients (16 out of 20) underwent amputation for complications of peripheral vascular disease (PVD), typically either critical ischemia in a non-salvageable limb, or failure of previous vascular reconstruction. Most of these cases (12 out of 16) were below-knee amputations (BKA); only four patients required above-knee amputation (AKA). Of the four non-PVD-related cases, BKA was performed in two patients for chronic pain following previous orthopedic surgery (49F, 50M), in one patient for chronic osteomyelitis refractory to antibiotic treatment (42F) and in a final patient (34M) who required bilateral amputation for acute ischemia (secondary to thromboembolism from infective endocarditis). Mean age at surgery was 67 years (range 34–92).

The choice of muscles was primarily dictated by the logistics and reproducibility of sampling, given the variation in level and quantity of discard material for each amputation, but with a view to including a range of muscle types (fast, slow, mixed). The original technique of motor point biopsy (for the peroneal muscles) as described by Coërs and Woolf (1959) was used as a guide. For most of the case series, we were able to obtain a complete set of samples from the four muscles chosen. In total, 72 individual muscles were analyzed (EDL = 19, S = 18, PB = 18, PL = 17).

Mice and Rats

To allow direct comparison with human NMJs, equivalent muscles were dissected from both sides of three CD1 littermate mice (adult, ~12 weeks old) and wild-type rats (adult, ~16 weeks old). Animals were euthanized with isoflurane and the muscles dissected out within 30 min post-mortem and fixed

in 4% PFA for 1 hr. All animal experiments were performed under the appropriate project and personal licenses granted by the UK Home Office.

Statistical Analysis

All statistical analyses were performed in GraphPad Prism. Individual statistical tests and significance levels are referred to in the relevant text sections and corresponding figure legends.

DATA AND SOFTWARE AVAILABILITY

The full raw proteomics data files from this study are freely available for download from: https://datashare.is.ed.ac.uk/handle/10283/2937.

SUPPLEMENTAL INFORMATION

Supplemental Information includes Supplemental Experimental Procedures, four figures, and three tables and can be found with this article online at https://doi.org/10.1016/j.celrep.2017.11.008.

AUTHOR CONTRIBUTIONS

R.A.J. and T.H.G. conceived and designed the study. R.A.J., C.H., C.S., S.L.E., T.M.W., and T.H.G. planned experiments. R.A.J., C.H., S.L.E., M.L.H., L.C.G., L.A., O.A.O., A.G., D.J.L., M.W.S., C.S., T.M.W., and T.H.G. performed experiments and data analysis. All authors drafted and approved the manuscript for submission.

ACKNOWLEDGMENTS

We are particularly grateful to Mr. Roderick Chalmers and the Vascular Department at the Royal Infirmary of Edinburgh for help in coordinating the tissue sampling and Frances Rae at Lothian NRS BioResource. This work was supported by small project grant funding from Biomedical Sciences (Anatomy) at the University of Edinburgh (T.H.G. and R.A.J.), the Darwin Trust of Edinburgh (M.L.H.), and the BBSRC (Institute Strategic Programme Funding; T.M.W., S.L.E., and L.C.G.).

Received: June 14, 2017
Revised: August 18, 2017
Accepted: November 1, 2017
Published: November 28, 2017

REFERENCES

Ackermann, F., Waites, C.L., and Garner, C.C. (2015). Presynaptic active zones in invertebrates and vertebrates. EMBO Rep. *16*, 923–938.

Anis, N.A., and Robbins, N. (1987). General and strain-specific age changes at mouse limb neuromuscular junctions. Neurobiol. Aging *8*, 309–318.

Balice-Gordon, R.J., and Lichtman, J.W. (1990). In vivo visualization of the growth of pre- and postsynaptic elements of neuromuscular junctions in the mouse. J. Neurosci. *10*, 894–908.

Coërs, C., and Woolf, A.L. (1959). The Innervation of Muscle (Oxford: Blackwell Scientific Publications).

Couteaux, R., and Pécot-Dechavassine, M. (1970). Synaptic vesicles and pouches at the level of "active zones" of the neuromuscular junction. C. R. Acad. Sci. Hebd. Seances Acad. Sci. D *271*, 2346–2349.

Fatt, P., and Katz, B. (1952). Spontaneous subthreshold activity at motor nerve endings. J. Physiol. *117*, 109–128.

Fukunaga, H., Engel, A.G., Lang, B., Newsom-Davis, J., and Vincent, A. (1983). Passive transfer of Lambert-Eaton myasthenic syndrome with IgG from man to mouse depletes the presynaptic membrane active zones. Proc. Natl. Acad. Sci. USA *80*, 7636–7640.

Gonzalez-Freire, M., de Cabo, R., Studenski, S.A., and Ferrucci, L. (2014). The neuromuscular junction: aging at the crossroad between nerves and muscle. Front. Aging Neurosci. *6*, 208.

Gonzalez-Freire, M., Semba, R.D., Ubaida-Mohien, C., Fabbri, E., Scalzo, P., Højlund, K., Dufresne, C., Lyashkov, A., and Ferrucci, L. (2017). The Human Skeletal Muscle Proteome Project: a reappraisal of the current literature. J. Cachexia Sarcopenia Muscle *8*, 5–18.

Han, J., Pluhackova, K., and Böckmann, R.A. (2017). The multifaceted role of SNARE proteins in membrane fusion. Front. Physiol. *8*, 5.

Huang, B., Babcock, H., and Zhuang, X. (2010). Breaking the diffraction barrier: super-resolution imaging of cells. Cell *143*, 1047–1058.

Jones, R.A., Reich, C.D., Dissanayake, K.N., Kristmundsdottir, F., Findlater, G.S., Ribchester, R.R., Simmen, M.W., and Gillingwater, T.H. (2016). NMJ-morph reveals principal components of synaptic morphology influencing structure-function relationships at the neuromuscular junction. Open Biol. *6*, 160240.

Kasthuri, N., Hayworth, K.J., Berger, D.R., Schalek, R.L., Conchello, J.A., Knowles-Barley, S., Lee, D., Vázquez-Reina, A., Kaynig, V., Jones, T.R., et al. (2015). Saturated reconstruction of a volume of neocortex. Cell *162*, 648–661.

Kay, K.R., Smith, C., Wright, A.K., Serrano-Pozo, A., Pooler, A.M., Koffie, R., Bastin, M.E., Bak, T.H., Abrahams, S., Kopeikina, K.J., et al. (2013). Studying synapses in human brain with array tomography and electron microscopy. Nat. Protoc. *8*, 1366–1380.

Liu, W., Klose, A., Forman, S., Paris, N.D., Wei-LaPierre, L., Cortés-Lopéz, M., Tan, A., Flaherty, M., Miura, P., Dirksen, R.T., and Chakkalakal, J.V. (2017). Loss of adult skeletal muscle stem cells drives age-related neuromuscular junction degeneration. eLife *6*, e26464.

Meinertzhagen, I.A., Govind, C.K., Stewart, B.A., Carter, J.M., and Atwood, H.L. (1998). Regulated spacing of synapses and presynaptic active zones at larval neuromuscular junctions in different genotypes of the flies Drosophila and Sarcophaga. J. Comp. Neurol. *393*, 482–492.

Murray, L.M., Talbot, K., and Gillingwater, T.H. (2010). Review: neuromuscular synaptic vulnerability in motor neurone disease: amyotrophic lateral sclerosis and spinal muscular atrophy. Neuropathol. Appl. Neurobiol. *36*, 133–156.

Nagwaney, S., Harlow, M.L., Jung, J.H., Szule, J.A., Ress, D., Xu, J., Marshall, R.M., and McMahan, U.J. (2009). Macromolecular connections of active zone material to docked synaptic vesicles and presynaptic membrane at neuromuscular junctions of mouse. J. Comp. Neurol. *513*, 457–468.

Newman, Z.L., Hoagland, A., Aghi, K., Worden, K., Levy, S.L., Son, J.H., Lee, L.P., and Isacoff, E.Y. (2017). Input-Specific Plasticity and Homeostasis at the Drosophila Larval Neuromuscular Junction. Neuron *93*, 1388–1404.e10.

Nishimune, H., Badawi, Y., Mori, S., and Shigemoto, K. (2016). Dual-color STED microscopy reveals a sandwich structure of Bassoon and Piccolo in active zones of adult and aged mice. Sci. Rep. *6*, 27935.

Nitkin, R.M., Smith, M.A., Magill, C., Fallon, J.R., Yao, Y.M., Wallace, B.G., and McMahan, U.J. (1987). Identification of agrin, a synaptic organizing protein from Torpedo electric organ. J. Cell Biol. *105*, 2471–2478.

Oda, K. (1984). Age changes of motor innervation and acetylcholine receptor distribution on human skeletal muscle fibres. J. Neurol. Sci. *66*, 327–338.

Oh, S.W., Harris, J.A., Ng, L., Winslow, B., Cain, N., Mihalas, S., Wang, Q., Lau, C., Kuan, L., Henry, A.M., et al. (2014). A mesoscale connectome of the mouse brain. Nature *508*, 207–214.

Qiu, J., McQueen, J., Bilican, B., Dando, O., Magnani, D., Punovuori, K., Selvaraj, B.T., Livesey, M., Haghi, G., Heron, S., et al. (2016). Evidence for evolutionary divergence of activity-dependent gene expression in developing neurons. eLife *5*, e20337.

Rogers, R.S., and Nishimune, H. (2017). The role of laminins in the organization and function of neuromuscular junctions. Matrix Biol. *57-58*, 86–105.

Shi, L., Fu, A.K., and Ip, N.Y. (2012). Molecular mechanisms underlying maturation and maintenance of the vertebrate neuromuscular junction. Trends Neurosci. *35*, 441–453.

Slater, C.R. (2015). The functional organization of motor nerve terminals. Prog. Neurobiol. *134*, 55–103.

Slater, C.R., Lyons, P.R., Walls, T.J., Fawcett, P.R.W., and Young, C. (1992). Structure and function of neuromuscular junctions in the vastus lateralis of

OPEN
ACCESS
CellPress

man. A motor point biopsy study of two groups of patients. Brain *115*, 451–478.

Valdez, G., Tapia, J.C., Kang, H., Clemenson, G.D., Jr., Gage, F.H., Lichtman, J.W., and Sanes, J.R. (2010). Attenuation of age-related changes in mouse neuromuscular synapses by caloric restriction and exercise. Proc. Natl. Acad. Sci. USA *107*, 14863–14868.

Wilhelm, B.G., Mandad, S., Truckenbrodt, S., Kröhnert, K., Schäfer, C., Rammner, B., Koo, S.J., Claßen, G.A., Krauss, M., Haucke, V., et al. (2014). Composition of isolated synaptic boutons reveals the amounts of vesicle trafficking proteins. Science *344*, 1023–1028.

Willadt, S., Nash, M., and Slater, C.R. (2016). Age-related fragmentation of the motor endplate is not associated with impaired neuromuscular transmission in the mouse diaphragm. Sci. Rep. *6*, 24849.

Wokke, J.H., Jennekens, F.G., van den Oord, C.J., Veldman, H., Smit, L.M., and Leppink, G.J. (1990). Morphological changes in the human end plate with age. J. Neurol. Sci. *95*, 291–310.

Cell Reports, Volume *21*

Supplemental Information

Cellular and Molecular Anatomy

# of the Human Neuromuscular Junction

Ross A. Jones, Carl Harrison, Samantha L. Eaton, Maica Llavero Hurtado, Laura C. Graham, Leena Alkhammash, Oladayo A. Oladiran, Andy Gale, Douglas J. Lamont, Hamish Simpson, Martin W. Simmen, Christian Soeller, Thomas M. Wishart, and Thomas H. Gillingwater

Figure S1

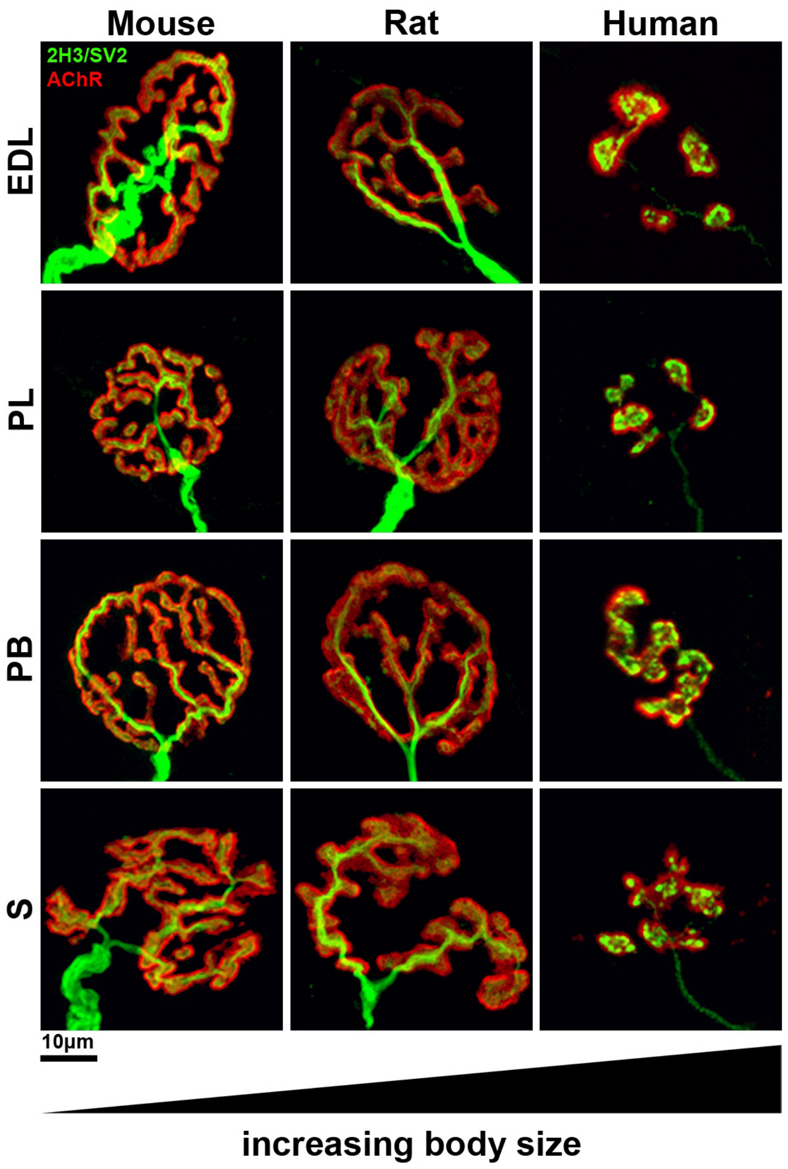

Figure S1 | Comparative anatomy of mouse, rat and human NMJs, Related to Figure 1.

Representative confocal micrographs of NMJs from equivalent muscles in the 3 different species, arranged from left to right in order of ascending body weight and size. There were no discernable differences between mouse and rat NMJs. Scale bar = 10 μm.

Figure S2

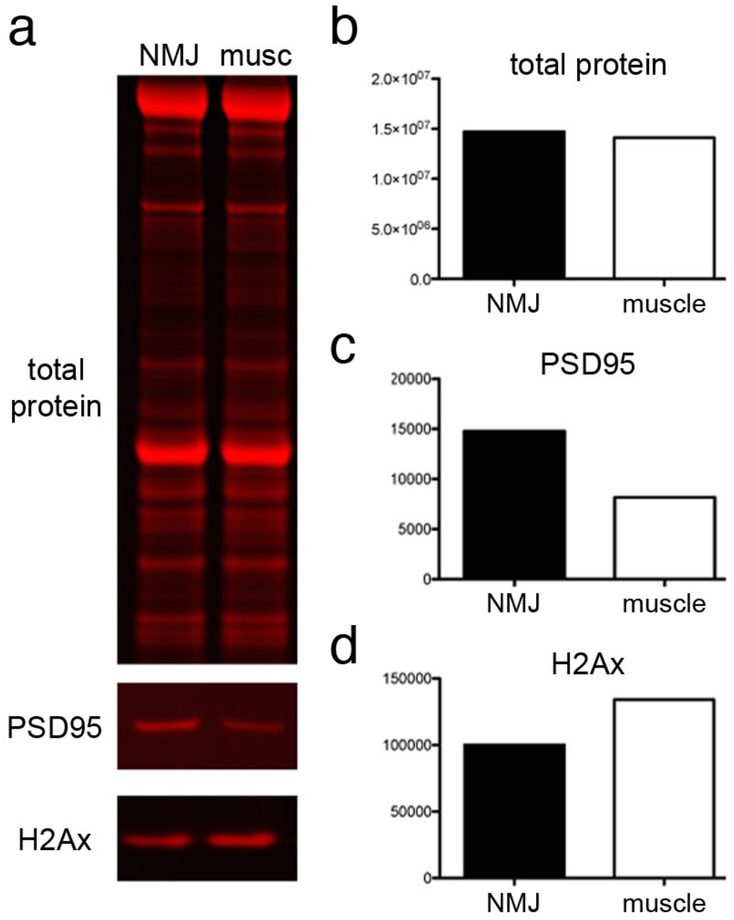

Figure S2 | Validation of NMJ-enrichment in human and mouse tissue, Related to Figure 4.

a. Samples enriched for NMJ content (NMJ) and devoid of NMJ content (muscle) were probed to confirm the efficacy of the isolation procedure used (see Methods and Figure 4). Representative LICOR captured images of gels stained for total protein load, and membranes probed for a post-synaptic marker (PSD95) and a nuclear marker (H2Ax) confirming **b.** equivalent protein extraction, **c.** enrichment for post-synaptic protein and **d.** de-enrichment for nuclei.

Figure S3

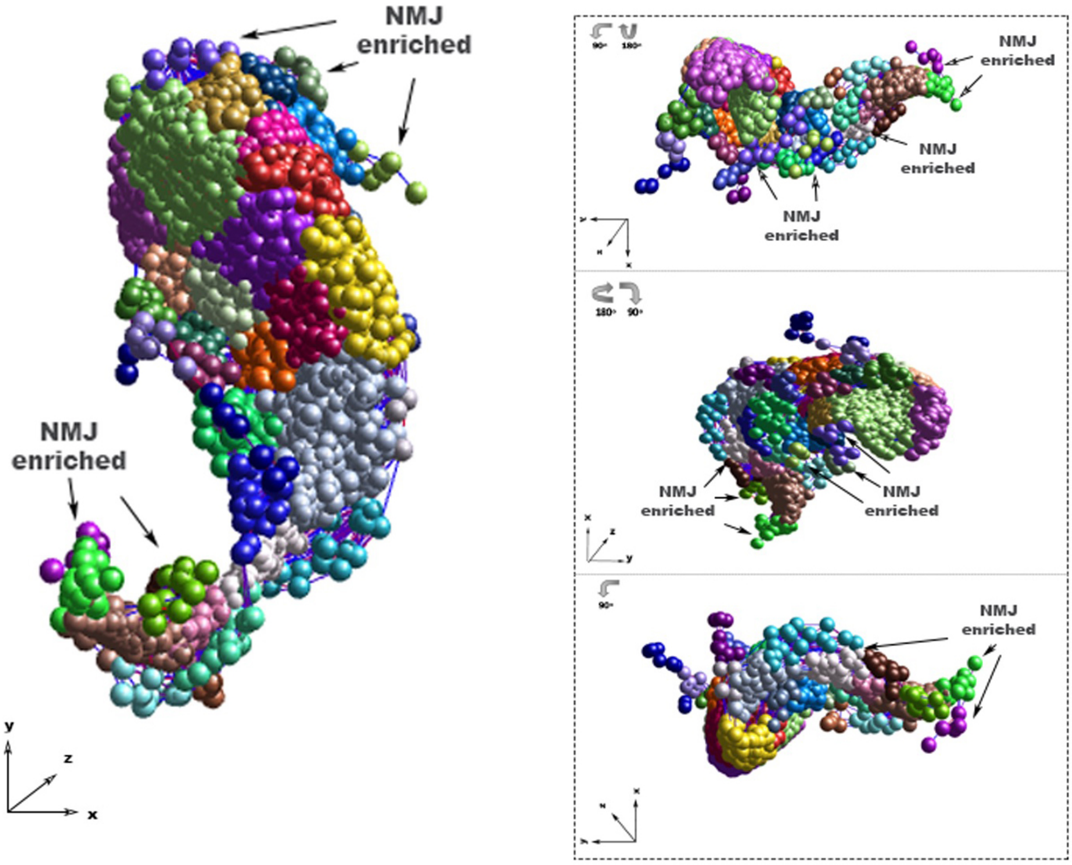

Figure S3 | Biolayout expression profile clustering identifies upregulated synaptic proteins in NMJ-enriched tissue samples, Related to Figure 4.

Nodes (spheres) represent individual proteins and edges (lines) reflect the strength of correlation (of expression) between proteins. Node colours represent distinct protein clusters based on their expression profile. The 3D schematic (shown in 4 different orientations) displays all proteins from both NMJ-enriched and NMJ-devoid human samples; the 5 distinct synaptic protein clusters from the NMJ-enriched samples are indicated with arrows.

Figure S4

Agrin interactions at the NMJ
(human vs mouse)

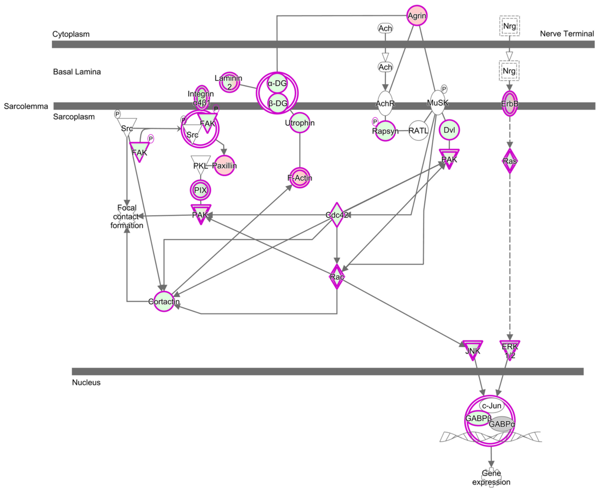

Axonal guidance signaling
(human vs mouse)

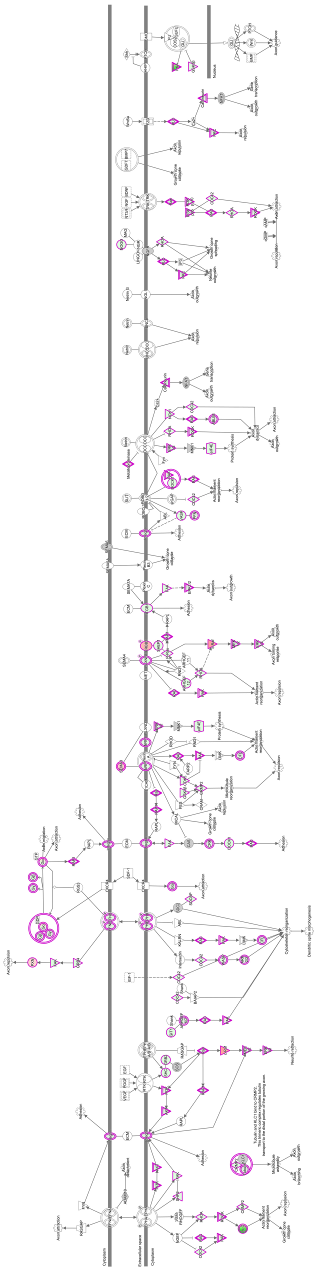

CREB signaling in neurons
(human vs mouse)

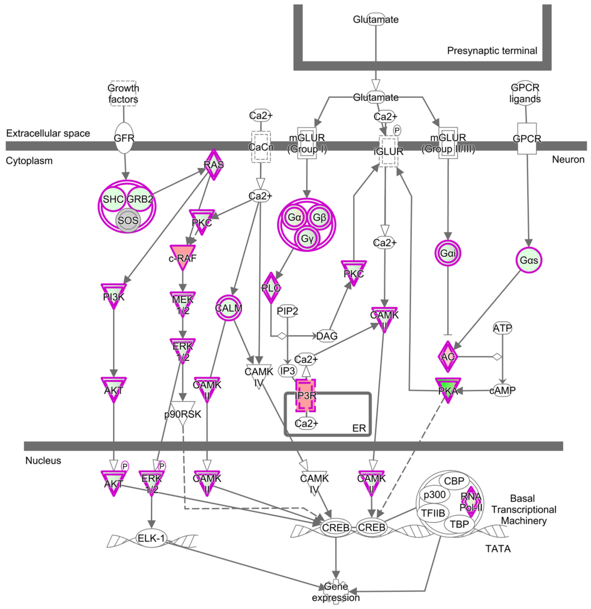

Figure S4 | Differences in protein composition between human and mouse NMJs, Related to Figure 4.

IPA canonical pathway analysis of proteomics data identified significant alterations in the molecular composition of NMJ-enriched samples from humans compared with mice. Several changes in core synaptic pathways such as agrin interactions at the NMJ (**a**), CREB signaling in neurons (**b**) and axonal guidance signaling (**c**) were identified. Nodes in red represent proteins more abundant in human samples relative to mouse (>20% increased in human); nodes in green represent proteins more abundant in mouse samples relative to human (>20% decreased in human); the darker the colour of the node, the greater the difference in protein expression. Grey nodes represent proteins that differ by <20% between human and mouse. Note that each pathway includes proteins that are both *more and less* abundant in human cf. mouse samples, indicating that this is not simply an artefact of relative enrichment during tissue processing.

Table S1

Table S1 Baseline morphological data and influence of pre- and post-synaptic cells

	average human NMJ	average mouse NMJ	fold difference	human NMJ: correlation with axon diameter	human NMJ: correlation with muscle fibre diameter	mouse NMJ: correlation with axon diameter	mouse NMJ: correlation with muscle fibre diameter
core varaibles							
pre-synaptic							
**1) nerve terminal area (µm^2)**	122.7	304.0	2.48 ****	0.548 ****	0.358 **	0.694 ***	0.099
**2) nerve terminal perimeter (µm)**	151.1	327.4	2.17 ****	0.435 **	0.408 **	0.429 *	0.273
**3) number of terminal branches**	28.0	30.0	1.07	0.248	0.355 **	0.370	0.016
**4) number of branch points**	14.0	26.0	1.86 ****	0.553 ****	0.250	0.609 **	-0.025
**5) total length of branches (µm)**	69.3	166.7	2.41 ****	0.533 ****	0.320 *	0.577 **	0.193
post-synaptic							
**6) AChR area (µm^2)**	206.7	424.2	2.05 ****	0.329 *	0.385 ***	0.572 **	0.076
**7) AChR perimeter (µm)**	152.2	275.8	1.81 ****	0.179	0.297 *	0.279	0.378
**8) endplate area (µm^2)**	351.5	678.2	1.93 ****	0.302 *	0.373 **	0.510 *	0.158
**9) endplate perimeter (µm)**	98.7	118.8	1.20 ****	0.363 **	0.373 **	0.521 **	0.060
**10) endplate diameter (µm)**	36.0	42.2	1.17 ***	0.350 *	0.344 **	0.421 *	0.069
**11) number of AChR clusters**	3.9	2.6	1.50 ****	0.225	0.151	0.306	0.181
derived variables							
pre-synaptic							
**12) average length of branches (µm)**	3.0	6.7	2.23 ****	0.522 ****	-0.146	-0.397	0.114
**13) complexity**	4.07	4.97	1.22 ****	0.424 **	0.275 *	0.582 **	0.064
post-synaptic							
**14) average area of AChR clusters (µm^2)**	71.7	238.5	3.33 ****	0.202	0.305 **	0.117	-0.085
**15) fragmentation**	0.58	0.40	1.45 ****	0.304 *	0.089	0.353	0.175
**16) compactness (%)**	61.6	64.4	1.05 **	0.000	-0.025	0.097	-0.357
**17) overlap (%)**	49.6	64.2	1.29 ****	0.382 **	-0.129	0.359	0.454 *
**18) area of synaptic contact (µm^2)**	105.2	267.9	2.55 ****	0.461 ***	0.370 **	0.660 ***	0.147
associated nerve & muscle variables							
**19) axon diameter (µm)**	0.84	3.10	3.69 ****				
**20) muscle fibre diameter (µm)**	59.9	40.2	1.49 ****				
**21) number of axonal inputs**	1	1	1.00				

Table S1 | Baseline morphological data and influence of pre- and post-synaptic cells, Related to Figure 1.

Core variables (1-11) are listed in red, derived variables (12-18) in blue and associated nerve and muscle variables (19-21) in green. The 'averages' listed (average human NMJ, average mouse NMJ) for each variable represent the mean for the complete set of NMJs (2860 human; 960 mouse). Fold difference (between human and mouse NMJ) is the ratio of the larger value relative to the smaller value; most NMJ variables (but not all) were larger in mice. Correlation data is listed as coefficient (*r*, numerical) and significance level (*p*, asterisk). [****p< 0.0001, ***p< 0.001, **p< 0.01, *p< 0.05]

Table S2

**Table S2** Patient case series							
case ref	age	sex	side	procedure	indication	DM	function
1R	34	m	R	BKA	non PVD	non DM	mobile
1L	34	m	L	BKA	non PVD	non DM	mobile
2	42	f	L	BKA	non PVD	non DM	mobile
3	49	f	R	BKA	non PVD	non DM	mobile
4	49	m	R	BKA	PVD	DM	mobile
5	50	m	R	BKA	non PVD	non DM	n/a
6	52	f	L	BKA	PVD	DM	n/a
7	56	f	L	BKA	PVD	DM	n/a
8	58	m	L	AKA	PVD	DM	mobile
9	64	m	R	BKA	PVD	DM	n/a
10	66	m	R	BKA	PVD	DM	mobile
11	68	m	R	BKA	PVD	DM	mobile
12	77	m	L	BKA	PVD	DM	n/a
13	79	m	R	BKA	PVD	DM	mobile
14	80	m	R	BKA	PVD	non DM	n/a
15	80	m	R	BKA	PVD	DM	mobile
16	84	m	L	AKA	PVD	DM	mobile
17	85	m	R	AKA	PVD	DM	n/a
18	89	m	L	AKA	PVD	non DM	n/a
19	92	f	L	BKA	PVD	non DM	mobile
20	92	m	L	BKA	PVD	non DM	n/a

Table S2 | Patient case series, Related to Figure 2.

Demographic and clinical details for the complete case series (20 patients). Age is in years. Abbreviations: m, male; f, female; R, right limb; L, left limb; BKA, below knee amputation; AKA, above knee amputation; PVD, peripheral vascular disease; DM, diabetes mellitus; n/a, data not available.

Table S3

Table S3 Human vs mouse muscle metabolic cascades

Ingenuity Canonical Pathways	$-\log_{10}$(*p* value)	Upregulated (H vs M)	Downregulated (H vs M)
Oxidative Phosphorylation	23.90	0/119 (0%)	75/119 (63%)
3-phosphoinositide Degradation	8.24	4/162 (2%)	64/162 (40%)
D-myo-inositol-5-phosphate Metabolism	8.18	4/166 (2%)	65/166 (39%)
D-myo-inositol (1,4,5,6)-tetrakisphosphate Biosynthesis	7.04	4/147 (3%)	57/147 (39%)
D-myo-inositol (3,4,5,6)-tetrakisphosphate Biosynthesis	7.04	4/147 (3%)	57/147 (39%)
tRNA Charging	6.49	2/82 (2%)	35/82 (43%)
3-phosphoinositide Biosynthesis	6.24	6/207 (3%)	69/207 (33%)
Glycolysis I	6.00	0/41 (0%)	22/41 (54%)
Superpathway of Inositol Phosphate Compounds	5.70	6/253 (2%)	79/253 (31%)
Ethanol Degradation II	5.34	1/47 (2%)	24/47 (51%)
Fatty Acid β-oxidation I	5.12	0/45 (0%)	23/45 (51%)
Gluconeogenesis I	4.93	1/46 (2%)	22/46 (48%)
Glutaryl-CoA Degradation	4.77	0/26 (0%)	15/26 (58%)
TCA Cycle II (Eukaryotic)	4.69	0/41 (0%)	20/41 (49%)
Oxidative Ethanol Degradation III	4.46	2/33 (6%)	18/33 (55%)
Ethanol Degradation IV	4.46	1/36 (3%)	19/36 (53%)
Fatty Acid α-oxidation	4.29	1/25 (4%)	15/25 (60%)
Glycogen Degradation III	4.15	0/20 (0%)	12/20 (60%)
Tryptophan Degradation X (Mammalian, via Tryptamine)	4.04	2/35 (6%)	18/35 (51%)
Glycogen Degradation II	3.94	0/18 (0%)	11/18 (61%)
Pyridoxal 5'-phosphate Salvage Pathway	3.67	1/75 (1%)	28/75 (37%)
Valine Degradation I	3.46	0/35 (0%)	16/35 (46%)
Glutathione Redox Reactions I	3.39	1/29 (3%)	14/29 (48%)
Noradrenaline and Adrenaline Degradation	3.24	1/57 (2%)	23/57 (40%)
D-myo-inositol (1,4,5)-trisphosphate Degradation	2.93	0/22 (0%)	11/22 (50%)
Isoleucine Degradation I	2.81	0/29 (0%)	13/29 (45%)
Histamine Degradation	2.70	1/33 (3%)	15/33 (45%)
Putrescine Degradation III	2.42	1/35 (3%)	15/35 (43%)
Superoxide Radicals Degradation	2.27	0/10 (0%)	6/10 (60%)
Salvage Pathways of Pyrimidine Ribonucleotides	2.19	1/112 (1%)	33/112 (29%)
Dopamine Degradation	2.08	1/49 (2%)	18/49 (37%)
Tryptophan Degradation III (Eukaryotic)	2.08	1/49 (2%)	17/49 (35%)
Thioredoxin Pathway	2.05	1/8 (13%)	5/8 (63%)
Glutathione Redox Reactions II	2.05	1/8 (13%)	5/8 (63%)
Calcium Transport I	2.02	0/14 (0%)	8/14 (57%)
Superpathway of Geranylgeranyldiphosphate Biosynthesi	1.97	1/35 (3%)	13/35 (37%)
Ketogenesis	1.88	0/18 (0%)	8/18 (44%)
Ketolysis	1.88	0/18 (0%)	8/18 (44%)
Colanic Acid Building Blocks Biosynthesis	1.86	1/36 (3%)	15/36 (42%)
Glutathione-mediated Detoxification	1.84	0/40 (0%)	15/40 (38%)
Pentose Phosphate Pathway	1.79	1/22 (5%)	9/22 (41%)
Acetyl-CoA Biosynthesis I (Pyruvate Dehydrogenase Comp	1.78	0/12 (0%)	6/12 (50%)
2-ketoglutarate Dehydrogenase Complex	1.77	0/9 (0%)	5/9 (56%)
Branched-chain α-keto acid Dehydrogenase Complex	1.77	0/9 (0%)	5/9 (56%)
Myo-inositol Biosynthesis	1.77	0/9 (0%)	5/9 (56%)
Guanosine Nucleotides Degradation III	1.65	0/23 (0%)	10/23 (43%)
Urate Biosynthesis/Inosine 5'-phosphate Degradation	1.65	0/23 (0%)	10/23 (43%)
Pyrimidine Ribonucleotides Interconversion	1.64	0/54 (0%)	18/54 (33%)
Mevalonate Pathway I	1.61	1/27 (4%)	10/27 (37%)
Stearate Biosynthesis I (Animals)	1.56	1/55 (2%)	18/55 (33%)
Pentose Phosphate Pathway (Oxidative Branch)	1.54	1/10 (10%)	5/10 (50%)
2-oxobutanoate Degradation I	1.50	0/17 (0%)	7/17 (41%)
Adenosine Nucleotides Degradation II	1.50	0/28 (0%)	11/28 (39%)
Folate Transformations I	1.50	1/28 (4%)	11/28 (39%)
Mitochondrial L-carnitine Shuttle Pathway	1.45	0/21 (0%)	9/21 (43%)
Methionine Degradation I (to Homocysteine)	1.41	0/25 (0%)	9/25 (36%)
D-myo-inositol (1,4,5)-Trisphosphate Biosynthesis	1.39	1/37 (3%)	11/37 (30%)
Phosphatidylglycerol Biosynthesis II (Non-plastidic)	1.39	3/41 (7%)	15/41 (37%)
Purine Nucleotides Degradation II (Aerobic)	1.39	0/37 (0%)	13/37 (35%)
Superpathway of D-myo-inositol (1,4,5)-trisphosphate M	1.39	0/33 (0%)	11/33 (33%)
Superpathway of Methionine Degradation	1.38	0/66 (0%)	19/66 (29%)
D-glucuronate Degradation I	1.35	1/11 (9%)	5/11 (45%)
Heme Degradation	1.35	1/11 (9%)	5/11 (45%)
Glucose and Glucose-1-phosphate Degradation	1.33	0/22 (0%)	9/22 (41%)
Pyrimidine Ribonucleotides De Novo Biosynthesis	1.32	0/67 (0%)	20/67 (30%)
Leucine Degradation I	1.31	0/26 (0%)	9/26 (35%)
NADH Repair	1.30	0/8 (0%)	4/8 (50%)
Glycerol-3-phosphate Shuttle	1.30	0/8 (0%)	4/8 (50%)
Hypusine Biosynthesis	1.28	0/5 (0%)	3/5 (60%)
CDP-diacylglycerol Biosynthesis I	1.22	3/35 (9%)	13/35 (37%)
Methylglyoxal Degradation III	1.22	1/23 (4%)	9/23 (39%)
NAD Salvage Pathway II	1.22	1/35 (3%)	12/35 (34%)
Serotonin Degradation	1.21	1/91 (1%)	25/91 (27%)
Methylmalonyl Pathway	1.19	0/12 (0%)	5/12 (42%)
NAD Phosphorylation and Dephosphorylation	1.13	1/20 (5%)	7/20 (35%)
Fatty Acid β-oxidation III (Unsaturated, Odd Number)	1.04	0/6 (0%)	3/6 (50%)
Retinoate Biosynthesis I	1.00	1/38 (3%)	11/38 (29%)
Acetate Conversion to Acetyl-CoA	0.96	0/10 (0%)	4/10 (40%)
Pyruvate Fermentation to Lactate	0.96	0/10 (0%)	4/10 (40%)
Tetrahydrofolate Salvage from 5,10-methenyltetrahydrof	0.96	0/10 (0%)	4/10 (40%)
Trans, trans-farnesyl Diphosphate Biosynthesis	0.96	0/10 (0%)	4/10 (40%)
Acyl-CoA Hydrolysis	0.94	0/14 (0%)	5/14 (36%)
Aspartate Degradation II	0.94	0/14 (0%)	5/14 (36%)
GDP-glucose Biosynthesis	0.93	0/18 (0%)	7/18 (39%)
Choline Degradation I	0.86	0/7 (0%)	3/7 (43%)
GDP-L-fucose Biosynthesis I (from GDP-D-mannose)	0.86	1/7 (14%)	3/7 (43%)
Proline Degradation	0.86	0/7 (0%)	3/7 (43%)
Thiosulfate Disproportionation III (Rhodanese)	0.86	0/7 (0%)	3/7 (43%)
Retinol Biosynthesis	0.86	2/49 (4%)	14/49 (29%)
Chondroitin Sulfate Degradation (Metazoa)	0.85	0/23 (0%)	7/23 (30%)
Heme Biosynthesis II	0.85	0/23 (0%)	7/23 (30%)
Sucrose Degradation V (Mammalian)	0.84	0/19 (0%)	6/19 (32%)
Superpathway of Serine and Glycine Biosynthesis I	0.84	0/19 (0%)	6/19 (32%)
Heme Biosynthesis from Uroporphyrinogen-III I	0.83	0/11 (0%)	4/11 (36%)
Phenylalanine Degradation I (Aerobic)	0.83	0/11 (0%)	4/11 (36%)
Phenylethylamine Degradation I	0.83	0/11 (0%)	4/11 (36%)
Rapoport-Luebering Glycolytic Shunt	0.83	0/11 (0%)	4/11 (36%)
Prostanoid Biosynthesis	0.83	1/15 (7%)	4/15 (27%)
Cysteine Biosynthesis III (mammalia)	0.82	0/32 (0%)	9/32 (28%)
Purine Nucleotides De Novo Biosynthesis II	0.81	0/41 (0%)	11/41 (27%)
Triacylglycerol Degradation	0.74	1/56 (2%)	14/56 (25%)
Superpathway of Citrulline Metabolism	0.73	2/38 (5%)	9/38 (24%)
Ascorbate Recycling (Cytosolic)	0.73	1/12 (8%)	4/12 (33%)
Biotin-carboxyl Carrier Protein Assembly	0.73	1/12 (8%)	3/12 (25%)
Glycogen Biosynthesis II (from UDP-D-Glucose)	0.73	0/12 (0%)	4/12 (33%)
N-acetylglucosamine Degradation II	0.73	0/12 (0%)	4/12 (33%)
NAD Biosynthesis III	0.73	0/12 (0%)	4/12 (33%)
S-methyl-5-thio-α-D-ribose 1-phosphate Degradation	0.73	0/12 (0%)	4/12 (33%)
Formaldehyde Oxidation II (Glutathione-dependent)	0.72	1/8 (13%)	3/8 (38%)
Geranylgeranyldiphosphate Biosynthesis	0.72	0/8 (0%)	3/8 (38%)
N-acetylglucosamine Degradation I	0.72	0/8 (0%)	3/8 (38%)
Retinoate Biosynthesis II	0.72	0/8 (0%)	3/8 (38%)
S-adenosyl-L-methionine Biosynthesis	0.72	0/8 (0%)	3/8 (38%)
Phospholipases	0.66	0/72 (0%)	17/72 (24%)
Triacylglycerol Biosynthesis	0.66	1/58 (2%)	16/58 (28%)
Adenine and Adenosine Salvage III	0.66	0/17 (0%)	5/17 (29%)
Lysine Degradation II	0.66	1/17 (6%)	5/17 (29%)
Citrulline Biosynthesis	0.65	2/26 (8%)	6/26 (23%)
UDP-N-acetyl-D-galactosamine Biosynthesis II	0.65	0/26 (0%)	9/26 (35%)
Arginine Degradation I (Arginase Pathway)	0.63	0/13 (0%)	4/13 (31%)
GDP-mannose Biosynthesis	0.63	0/13 (0%)	5/13 (38%)
Molybdenum Cofactor Biosynthesis	0.63	0/13 (0%)	4/13 (31%)
Pentose Phosphate Pathway (Non-oxidative Branch)	0.63	0/13 (0%)	4/13 (31%)
Adenine and Adenosine Salvage I	0.61	0/9 (0%)	3/9 (33%)
Creatine-phosphate Biosynthesis	0.61	0/9 (0%)	3/9 (33%)
Guanine and Guanosine Salvage I	0.61	0/9 (0%)	3/9 (33%)
The Visual Cycle	0.57	1/32 (3%)	9/32 (28%)
Galactose Degradation I (Leloir Pathway)	0.55	0/14 (0%)	5/14 (36%)
Proline Biosynthesis I	0.55	2/14 (14%)	3/14 (21%)
Serine Biosynthesis	0.55	0/14 (0%)	4/14 (29%)
Fatty Acid Activation	0.52	0/19 (0%)	6/19 (32%)
4-aminobutyrate Degradation I	0.52	0/10 (0%)	3/10 (30%)
Thymine Degradation	0.52	1/10 (10%)	3/10 (30%)
Arginine Biosynthesis IV	0.50	1/24 (4%)	6/24 (25%)
γ-linolenate Biosynthesis II (Animals)	0.50	0/24 (0%)	7/24 (29%)
γ-glutamyl Cycle	0.49	1/29 (3%)	8/29 (28%)
Glycine Biosynthesis I	0.48	0/6 (0%)	2/6 (33%)
S-methyl-5'-thioadenosine Degradation II	0.48	0/6 (0%)	2/6 (33%)
Purine Ribonucleosides Degradation to Ribose-1-phospha	0.47	0/20 (0%)	5/20 (25%)
Glutathione Biosynthesis	0.44	1/11 (9%)	3/11 (27%)
Uracil Degradation II (Reductive)	0.44	1/11 (9%)	3/11 (27%)
Arginine Degradation VI (Arginase 2 Pathway)	0.43	1/16 (6%)	4/16 (25%)
Citrulline-Nitric Oxide Cycle	0.43	1/16 (6%)	4/16 (25%)
Inosine-5'-phosphate Biosynthesis II	0.43	0/16 (0%)	4/16 (25%)
Selenocysteine Biosynthesis II (Archaea and Eukaryotes)	0.43	0/16 (0%)	4/16 (25%)
D-myo-inositol (1,3,4)-trisphosphate Biosynthesis	0.41	0/26 (0%)	6/26 (23%)
Pyrimidine Deoxyribonucleotides De Novo Biosynthesis I	0.40	0/41 (0%)	9/41 (22%)
Aspartate Biosynthesis	0.39	0/7 (0%)	2/7 (29%)
Glutamate Biosynthesis II	0.39	1/7 (14%)	2/7 (29%)
Glutamate Degradation X	0.39	1/7 (14%)	2/7 (29%)
Glutamine Biosynthesis I	0.39	0/7 (0%)	2/7 (29%)
Tyrosine Biosynthesis IV	0.39	0/7 (0%)	2/7 (29%)
UDP-D-xylose and UDP-D-glucuronate Biosynthesis	0.39	0/7 (0%)	2/7 (29%)
Glutamate Degradation III (via 4-aminobutyrate)	0.38	0/12 (0%)	3/12 (25%)
CMP-N-acetylneuraminate Biosynthesis I (Eukaryotes)	0.37	0/17 (0%)	4/17 (24%)
UDP-N-acetyl-D-glucosamine Biosynthesis II	0.37	0/17 (0%)	4/17 (24%)
Choline Biosynthesis III	0.37	0/22 (0%)	5/22 (23%)
Phenylalanine Degradation IV (Mammalian, via Side Chai	0.37	1/32 (3%)	7/32 (22%)
Superpathway of Cholesterol Biosynthesis	0.36	1/83 (1%)	17/83 (20%)
1D-myo-inositol Hexakisphosphate Biosynthesis II (Mamr	0.33	0/28 (0%)	6/28 (21%)
Dermatan Sulfate Degradation (Metazoa)	0.33	0/23 (0%)	5/23 (22%)
Lysine Degradation V	0.33	1/23 (4%)	5/23 (22%)
Folate Polyglutamylation	0.33	0/18 (0%)	4/18 (22%)
Glycerol Degradation I	0.32	0/13 (0%)	3/13 (23%)
Glycine Cleavage Complex	0.32	0/13 (0%)	3/13 (23%)
L-carnitine Biosynthesis	0.32	0/13 (0%)	3/13 (23%)
NAD Salvage Pathway III	0.32	0/13 (0%)	3/13 (23%)
Tetrapyrrole Biosynthesis II	0.32	0/13 (0%)	3/13 (23%)
Alanine Biosynthesis III	0.32	0/3 (0%)	1/3 (33%)
D-mannose Degradation	0.32	0/3 (0%)	1/3 (33%)
Glutamate Removal from Folates	0.32	0/3 (0%)	1/3 (33%)
Lanosterol Biosynthesis	0.32	0/3 (0%)	1/3 (33%)
Acetyl-CoA Biosynthesis III (from Citrate)	0.32	0/8 (0%)	2/8 (25%)
Flavin Biosynthesis IV (Mammalian)	0.32	0/8 (0%)	2/8 (25%)
Methylglyoxal Degradation I	0.32	0/8 (0%)	2/8 (25%)
PRPP Biosynthesis I	0.32	0/8 (0%)	2/8 (25%)
Xanthine and Xanthosine Salvage	0.32	0/8 (0%)	2/8 (25%)
Proline Biosynthesis II (from Arginine)	0.29	1/19 (5%)	4/19 (21%)
dTMP De Novo Biosynthesis	0.28	1/14 (7%)	3/14 (21%)
1,25-dihydroxyvitamin D3 Biosynthesis	0.27	1/9 (11%)	2/9 (22%)
Cardiolipin Biosynthesis II	0.27	0/9 (0%)	2/9 (22%)
Epoxysqualene Biosynthesis	0.27	1/9 (11%)	2/9 (22%)
Glutamate Degradation II	0.27	0/9 (0%)	2/9 (22%)
Glycine Degradation (Creatine Biosynthesis)	0.27	0/9 (0%)	2/9 (22%)
L-cysteine Degradation III	0.27	0/9 (0%)	2/9 (22%)
Methylglyoxal Degradation VI	0.27	0/9 (0%)	2/9 (22%)
Oxidized GTP and dGTP Detoxification	0.27	0/9 (0%)	2/9 (22%)
Urea Cycle	0.25	0/20 (0%)	4/20 (20%)
Acyl Carrier Protein Metabolism	0.24	0/4 (0%)	1/4 (25%)
Sulfite Oxidation IV	0.24	0/4 (0%)	1/4 (25%)
Trehalose Degradation II (Trehalase)	0.22	0/10 (0%)	3/10 (30%)
β-alanine Degradation I	0.22	0/10 (0%)	2/10 (20%)
Adenine and Adenosine Salvage VI	0.18	0/5 (0%)	1/5 (20%)
Anandamide Degradation	0.18	0/5 (0%)	1/5 (20%)
Asparagine Degradation I	0.18	0/5 (0%)	1/5 (20%)
Glutamine Degradation I	0.18	0/5 (0%)	1/5 (20%)
Melatonin Degradation III	0.18	0/5 (0%)	1/5 (20%)
Methylthiopropionate Biosynthesis	0.18	0/5 (0%)	1/5 (20%)
Sorbitol Degradation I	0.18	0/5 (0%)	1/5 (20%)

Table S3 | Conservation of metabolic cascades between human and mouse muscle proteomes, Related to Figure 4.

Out of the 200 metabolic pathways identified in-silico, only 68 show differential proteomic expression between the species (pathways with a $-\log_{10}(p \text{ value}) \geq 1.3$). 66% of the known metabolic cascades are therefore unchanged between human and mouse muscle (NMJ-devoid) samples. Table layout and abbreviations are the same as in Figure 4 (panel d).

Supplemental Experimental Procedures:

Tissue harvesting

Muscle samples were obtained from the amputation specimen in the operating theatre immediately after disconnection of the limb. Tissue was harvested from the proximal end of the specimen, close to the line of surgical incision, in areas demonstrating good back bleeding and muscle fasciculation, and away from areas of necrosis and infection. Any muscle that appeared obviously devitalized on gross inspection was not sampled.

Small blocks of tissue, containing full-length muscle fibres from origin to insertion (approx. 2cm in length) were removed from each of the muscles selected and either immediately fixed in 4% paraformaldehyde for 1 hour or placed on wet ice (depending on the subsequent analyses, see below). All specimens were then transferred from theatre to laboratory for immediate processing.

The major concern relating to the use of tissue harvested from amputation specimens is the potential effect of chronic tissue ischaemia and diabetes mellitus (DM). Although twelve of the twenty patients had one or other form of diabetes, the functional status of all patients (where documented) was commensurate with standing and some degree of walking prior to surgery (with one 92-year-old female able to walk half a mile; Table S2).

At surgery, the level of amputation is principally determined by the likelihood of wound healing; a more distal amputation is the preferred option from a functional point of view, but not at the expense of healing. For the amputation stump to heal

fully the local blood supply must be adequate; thus, tissue sampled close to the line of incision (level of amputation) should also be of adequate health. The final decision on the level of amputation is determined during the procedure itself, by assessing the degree of back bleeding from the stump after disconnection of the limb. If the tissues are clearly devitalized, the amputation is immediately revised to a level at which these conditions are satisfied. These basic principles, along with the frequency with which lower limb amputation is performed (over 4,000 per year in England from 2003-2009) (Ahmad et al., 2014), provided the rationale for utilizing this method of tissue sampling. In addition, all samples were harvested from muscle that demonstrated visible contraction during dissection.

No significant differences were found in mean muscle fibre diameter when comparing patients with and without PVD (Figure 1), excluding any significant sarcopenia and/or disuse atrophy in the present case series. Furthermore, no significant differences were noted in the majority of the core morphological variables measured, when comparing NMJs in patients with or without either PVD (n.s. for 8 of 11 variables) or diabetes (n.s. for 10 of 11) (Figure 1). In addition, the general appearance of NMJs on visual inspection was remarkably conserved even when comparing NMJs at the extremes of the case series (young, non-PVD cf. elderly, PVD; Figure 2).

Muscle dissection and NMJ immunohistochemistry

Following muscle harvest, both human and rodent tissue was processed in an identical manner. Small bundles of 25-30 muscle fibres were teased out from the larger blocks/whole muscles, and of a size suitable for whole-mount preparation. NMJs

were then immunolabelled for presynaptic 2H3/SV2 proteins and postsynaptic AChRs according to the following protocol (antibodies and concentrations are listed below): α-bungarotoxin for 30 minutes; 4% Triton X for 90 minutes; 'block' for 30 minutes (4% bovine serum albumin and 2% Triton X); primary antibodies (in block) for 3 nights at 4°C; 4 washes of 1xPBS; secondary antibodies for 1 night at 4°C; 4 washes of 1xPBS. Preparations were then whole-mounted in Mowiol and stored at -20°C prior to imaging.

Antibodies

Primary antibodies: 1:50 mouse anti-2H3 IgG and 1:50 mouse anti-SV2 IgG (Developmental Studies Hybridoma Bank). Secondary antibodies: 1:50 Alexa Fluor 488 donkey anti-mouse IgG (Thermo Fisher Scientific). 1:500 TRITC α-bungarotoxin (Biotium).

Confocal imaging and NMJ-morph analysis

Images were acquired and analyzed using a standardized workflow, 'NMJ-morph', as previously described (Jones et al., 2016). Briefly, a Zeiss LSM 710 confocal microscope was used to acquire z-stack projections of individual en-face NMJs, including a short length of their pre-terminal axon. Images were then analyzed according to the described workflow, which uses ImageJ or Fiji (and freely availably plugins) to measure 21 individual pre- and post-synaptic morphological variables. Again, based on the NMJ-morph recommendations, 40 NMJs per muscle were analyzed to achieve accuracy of reported mean values. In total, we analyzed 2,860 individual human NMJs and 960 comparative mouse NMJs.

Muscle fibre diameters

Following confocal microscopy, the teased preparations were re-imaged at x20 magnification using an Olympus IX71 microscope, Hamamatsu C4742-95 camera and Openlab Improvision software. Measurement of muscle fibre diameter was performed manually in ImageJ; again, 40 individual fibres were analyzed per muscle (3,820 fibres in total for the complete human/mouse dataset).

dSTORM super-resolution imaging and SNAP25 quantification

A subset of peroneus longus muscle fibres was selected from three human cases. For comparison, an equivalent set of PL muscle fibres were obtained from three CD1 mice. Muscle dissection and immunohistochemistry was performed as described above, with the following modifications: after incubation in the primary antibodies (rabbit anti-SNAP25 IgG, 1:100; Alomone) for 3 nights at 4°C, tissue samples were labeled with Alexa Fluor 647 goat anti-rabbit IgG (1:100; Thermo Fisher Scientific) at room temperature for 2 hours. Specimens were then washed and mounted in dSTORM mounting medium (90% glycerol, 1xPBS, 100mM MEA) (Hou et al., 2015). Human samples underwent an additional 70-minute treatment with 1mM CuSO4 in 50mM ammonium acetate buffer, pH5 (Schnell et al., 1999) to remove lipofuscin-genic autofluorescence after immunolabelling.

Imaging was performed on a customized Nikon Ti Eclipse inverted fluorescent TIRF microscope with a 642nm laser. The lateral stage control was electronic and a piezo focuser (P-725, Physik Instrumente, Germany) precisely controlled the axial position. 30,000-65,000 frames were recorded with a 25ms acquisition time, in a z-step format. Z-steps were repeatedly performed at z-intervals of 200nm. Z-limits were set

manually, slightly above and below the plane of interface between nerve terminal and motor endplate. Only switching events in the given focal plane were accepted by a thresholding filter as part of the PYME implemented jittered triangulation algorithm (Hou et al., 2015). The same settings were used to capture a 550nm LED illuminated widefield stack of TRITC-α-bungarotoxin-labeled AChRs through a Texas Red filter set prior to dSTORM imaging. AChR stacks were used to produce a mean intensity projection image to act as a mask.

Quantification of SNAP25 distribution was performed in an identical manner for all images, using ImageJ. The 'masked' images (SNAP25/AChR overlays) were used as a reference when determining the boundaries of individual boutons/SNAP25 puncta on the dSTORM image, from which all measurements were made. dSTORM images of complete NMJs were first cropped to produce a series of images, each centred on an individual bouton (human) or section of bouton (mouse). The cropped dSTORM images were then converted into 8 bit grayscale counterparts, from which the mean gray value ('intensity') could be measured. These grayscale images were then converted into binary counterparts (using default thresholding) for the subsequent quantification of puncta. Binary images produced by default thresholding provided accurate representations of the original dSTORM images (i.e. no manual adjustments or other thresholding algorithms were required), and this approach was applied consistently for all images. Images were then 'despeckled' to sharpen the edges of individual puncta, and used to quantify the remaining variables: the average area of individual puncta and their density within each bouton, and the total area of all puncta relative to that of each bouton. The derived variable 'total area of active zones per NMJ' (Figure 3) was calculated by multiplying the latter variable (area of puncta :

area of bouton, dSTORM data) by the mean area of synaptic contact for NMJs in peroneus longus (baseline morphological data). Overall, we quantified 50 boutons (n) from 10 NMJs, for 3 individual patients/mice (N) – a total of 2,945 (human) and 10,666 (mice) individual puncta.

Proteomics: tissue sampling

For proteomic analysis, a further subset of PL muscle fibres was selected from four human cases. An equivalent set of PL muscle fibres was obtained from four CD1 mice for comparison. After tissue harvest, muscle dissection was performed on unfixed tissue, in a manner otherwise similar to that described above, to obtain small bundles of 25-30 muscle fibres. In order to identify the location of the endplate bands, muscle fibres were labelled with α-bungarotoxin for 5 minutes; the NMJ-enriched portions of the muscle fibres were then micro-dissected under a Nikon Eclipse 50i fluorescence microscope (Figure 4). NMJ-enriched samples, along with NMJ-devoid muscle 'ends', were then frozen to -80°C prior to proteomic analysis. All muscle dissection and labelling was performed within 30 minutes post-harvest to limit the degree of protein degradation.

Proteomics: protein extraction

Dissected tissue samples were pooled in M tubes (gentle MACS Miltenyi Biotec). Samples were homogenized in label-free extraction buffer [100 mM Tris-HCl (pH7.6) 4% (w/v) SDS] containing 1% protease cocktail inhibitor (Thermo Fisher, UK) using gentle MACS dissociator (Miltenyi Biotec) run on the M tube protein cycle. Post homogenization samples were spun at 300 x g for 2 minutes and left on ice for 20 minutes. Homogenates were transferred to Lo-Bind 1.5ml tubes (Sigma Aldrich) and

centrifuged at 20,000 x g for 20 minutes at 4°C with the soluble fraction of each sample then transferred to new Lo-Bind tubes. Protein determination using the Bicinchoninic acid assay (Pierce, UK) was carried out according to manufacturer's guidelines.

Proteomics: mass spectrometry

Preparation of samples, quantification and bioinformatics was performed according to a standardized protocol (Hughes et al., 2014). Samples were measured out to achieve 200ug/ul protein. DTT was added to give a 10mM final concentration and samples were left on a shaker at room temperature for 30 minutes, followed by incubation with IAA (50mM final concentration) in the dark for 30 minutes. 2ul DTT (1M) was added before samples were frozen.

Hydrophobic and hydrophilic beads (GE HealthCare) were mixed together and washed with MQ water. Beads were transferred to 4 tubes. Samples were then gently added to the beads and mixed/shaken for 2 min. 1% FA was then added to each sample (2 min mix), followed by 50% ACN (8 min mix); samples were then transferred to a magnetic rack and the supernatant was removed. Next, 70% EtOH was added (1 min mix), followed by removal of the supernatant. Finally, ACN was added (1 min mix) and the supernatant again removed.

Proteins were eluted with 100mM TEAB on a shaker for 10 min. Trypsin in 1mM HCl (1ug/ul) was added to each sample and incubated on a shaker at 30°C for 4-6h. A further portion of Trypsin was added and the samples were incubated overnight at 30°C. The tryptic peptides were then labelled with 6-plex TMT reagents (Thermo

Fisher Scientific) using a protocol supplied by the manufacturer [TMT6plex-Nter126 & -Lys126 – Human +, TMT6plex-Nter127 & -Lys127 – Human -, TMT6plex-Nter128 & -Lys128 – Mouse +, TMT6plex-Nter129 & -Lys129 – Mouse -]. The labelled peptides were quenched then mixed together.

The mixed sample was fractionated into 22 fractions using High pH reverse phase chromatography (Ultimate 3000 from Dionex). HPLC buffer A was 10mM ammonium formate in water (pH=10); HPLC buffer B was 10mM ammonium formate in 90% CH_3CN (pH=10). An XBridge peptide BEH column (130Å, 3.5 μm 2.1 X 150 mm from Waters) was used to separate peptides, with the column temperature set to 20°C. Peptides were eluted from the column using a flow rate of 200ul/min and a linear gradient of 5% to 60% buffer B in 60min. 40 fractions were collected and concatenated into 22 fractions based upon UV signal. All fractions were vacuumed dried and re-suspended in 50 μl 1%FA acid prior to MS analysis.

Fractions were analyzed on a Q-exactive-HF mass spectrometer (Thermo Scientific) equipped with Dionex Ultimate 3000 RS and Easyspray column (75 µm x 50 cm, PepMap RSLC C18 column, 2 µm, 100 Å). LC buffer A was 0.1% formic acid in MS grade Milli-Q water; LC buffer B was 80% acetonitrile and 0.08% formic acid in MS grade Milli-Q water. The peptides were eluted from the column using a flow rate of 300nl/min and a linear gradient of 5% to 40% buffer B in 122 min. The column temperature was set to 50°C.

Qexactive HF was performed in data dependent mode: an MS survey scan followed by 15 sequential dependent MS2 scans, with the 15 most intense precursor ions

selected to be fragmented by Higher Energy Collisional Dissociation (HCD), with the isolation window at 0.4 da. The resolution of the MS1 and MS2 was set at 120,000 and 60,000 respectively. The maximum ion injection time for MS1 and MS2 was 50ms and 200ms respectively.

Proteomics: quantification and bioinformatics analysis

The raw mass spectrometric data files obtained for each experiment were collated into a single quantitated dataset using MaxQuant (Cox and Mann, 2008) and Andromeda search engine software (Cox et al., 2011). Enzyme specificity was set to that of trypsin, allowing for cleavage of N-terminal to proline residues and between aspartic acid and proline residues. Other parameters used were: (i) variable modifications, deamidation (NQ), oxidation (M), protein N-acetylation, gln → pyro-glu; (ii) fixed modifications, carbamidomethylation (C); (iii) database: uniprot-reviewed_Mus_A10090_160916 & uniprot-Human-up5640_160516; (iv) Reporter ion MS2 – 4 TMT labels: TMT6plex-Nter126 to 129 & TMT6plex-Lys126 to 129; (v) MS/MS tolerance: FTMS- 10ppm, ITMS- 0.02 Da; (vi) maximum peptide length, 6; (vii) maximum missed cleavages, 2; (viii) maximum of labelled amino acids, 3; and (ix) false discovery rate, 1%. Peptide ratios were calculated using 'Reporter Intensity' for Mouse +/- samples, Human +/- samples and Human/Mouse +/- samples. Data was normalised using 1/median ratio value for each identified protein group per labelled sample. Proteins were filtered to include candidates identified by >1 peptide and demonstrating a >20% change between species. Filtered data was utilised for all bioinformatics statistical analyses including Biolayout Express 3D and Ingenuity Pathway Analyses (IPA). Expression clustering was performed in Biolayout Express 3D software by applying Markov clustering algorithms to raw proteomic data (MCL

2.2). All graphs were clustered using Pearson correlation r=0.96. IPA was performed as previously described (Wishart et al., 2007) with the interaction data limited as follows: direct and indirect interactions; experimentally observed data only; 35 molecules per network; 10 networks per dataset. Prediction activation scores (z-scores) were calculated in IPA.

<u>Proteomics: validation of synaptic enrichment</u>

Human and mouse pooled samples (10 μg load) were separated by SDS-polyacrylamide gel electrophoresis on 4–12% precast NuPage Bis-Tris gradient gels (Life technologies, UK) and then transferred to PVDF membrane using an iBlot 2 fast transfer device (Life Technologies, UK). The membranes were then blocked using Odyssey blocking buffer (Li-cor Biosciences, UK) and incubated with primary antibodies according to the manufacturers' instructions. Secondary antibodies (goat anti-rabbit IRDye 680 and donkey anti-mouse IRDye 680, Li-cor Biosciences, UK) were added according to the manufacturers' instructions. Blots were imaged using an Odyssey Infrared Imaging System (Li-cor Biosciences, UK). Scan resolution of the instrument ranged from 21 to 339 μm, and blots were imaged at 169 μm. Quantification was performed on single channels with the analysis software provided. Total protein stained gels were loaded in parallel with those used for membrane transfer. Gels were stained with InstantBlue (Expedeon) to ensure equal sample loading and were analysed using the Odyssey Infrared Imaging system as previously described (Eaton et al., 2013).

Supplemental References:

Ahmad, N., Thomas, G.N., Gill, P., Chan, C., and Torella, F. (2014). Lower limb amputation in England: prevalence, regional variation and relationship with revascularisation, deprivation and risk factors. A retrospective review of hospital data. J. Royal Soc. Med. *107*, 483-489.

Cox, J., and Mann, M. (2008). MaxQuant enables high peptide identification rates, individualized p.p.b.-range mass accuracies and proteome-wide protein quantification. Nat. Biotechnol. *26*, 1367-1372.

Cox, J., Neuhauser, N., Michalski, A., Scheltema, R.A., Olsen, J.V., and Mann, M. (2011). Andromeda: A Peptide Search Engine Integrated into the MaxQuant Environment. J. Proteome Res. *10*, 1794-1805.

Eaton, S.L., Roche, S.L., Hurtado, M.L., Oldknow, K.J., Farquharson, C., Gillingwater, T.H., and Wishart, T.M. (2013). Total Protein Analysis as a Reliable Loading Control for Quantitative Fluorescent Western Blotting. PLoS One *8*, doi: 10.1371/journal.pone.0072457.

Hou, Y., Jayasinghe, I., Crossman, D.J., Baddeley, D., and Soeller, C. (2015). Nanoscale analysis of ryanodine receptor clusters in dyadic couplings of rat cardiac myocytes. J. Mol. Cell. Cardiol. *80*, 45-55.

Wishart, T.M., Paterson, J.M., Short, D.M., Meredith, S., Robertson, K.A., Sutherland, C., Cousin, M.A., Dutia, M.B., and Gillingwater, T.H. (2007). Differential proteomics

analysis of synaptic proteins identifies potential cellular targets and protein mediators of synaptic neuroprotection conferred by the slow Wallerian degeneration (Wld^s) gene. Mol. Cell. Proteomics *6*, 1318-1330.
